# Supplementary material for: Microbiological quality assessment of potential pathogenic bacteria and multidrug resistance patterns in commercial electrolyte drinks in Dhaka, Bangladesh
Source: PLoS One. 2026 Jun 2;21(6):e0336888. doi: 10.1371/journal.pone.0336888 (PMC13229343; doi:10.1371/journal.pone.0336888)
Supplement: S1 Table — This table lists the kinds of media that are used to grow a certain kind of bacteria, along with the anticipated morphological colonies that will be observed on those media. (PDF) [file pone.0336888.s001.pdf]

Supporting information

Organism Name	Gram (+ve/-ve)	Media	Expected Colony Morphology
Klebsiella pneumoniae	Gram-negative	MacConkey Agar	Rod shaped, pink to dark pink colonies
		HiCrome UTI Agar, Modified	blue to purple
Escherichia coli	Gram-negative	XLD Agar	Rod shaped, yellow colonies
		HiCrome UTI Agar, Modified	Purple to magenta
Staphylococcus aureus	Gram-positive	MSA Agar	Cocci shaped, yellow/white colonies surrounded by a yellow zone
Staphylococcus epidermidis	Gram-positive	MSA Agar	Cocci shaped, grape-like cluster, red
Pseudomonas aeruginosa	Gram-negative	Pseudomonas Cetrimide Agar	Rod shaped, yellow-green glow under UV rays
Listeria spp.	Gram-positive	Listeria Selective Oxford Agar	Rod shaped, positive reaction for esculin hydrolysis, blackening of the medium around the colony
Acinetobacter baumannii	Gram-negative	Leeds Acinetobacter Agar Base	Short, almost round, rod shaped, pink mucoid colonies with pink color diffused into the medium
Vibrio spp.	Gram-negative	TCBS Agar	Curved rod (comma shaped) yellow/green colonies

S1 Table. Colony Morphology of Specific Bacteria on Selective Media.
