# Supplementary material for: Microbiological quality assessment of potential pathogenic bacteria and multidrug resistance patterns in commercial electrolyte drinks in Dhaka, Bangladesh
Source: PLoS One. 2026 Jun 2;21(6):e0336888. doi: 10.1371/journal.pone.0336888 (PMC13229343; doi:10.1371/journal.pone.0336888)
Supplement: S2 Table — It explains the kind of antibiotic group, its potency, spectrum, and interpreting standards. (PDF) [file pone.0336888.s002.pdf]

Supporting information

Antibiotic Class	Antibiotic (Disc Content in µg)	Effective against	Interpretative Criteria		
			Sensitive (S)	Intermediate (I)	Resistant (R)
Beta-lactamase	Ampicillin (AMP10)	Gram-positive and Gram-negative	17	14-16	13
	Amoxicillin (AM30)	Gram-positive and Gram-negative	17	14-16	13
	Amoxiclav (AMC30)	Gram-positive and Gram-negative	18	14-17	13
Cephalosporin	Cefoxitin (FOX30)	Gram-positive and Gram-negative	18	15-17	14
	Cefotaxime (CTX30)	Gram-positive and Gram-negative	26	23-25	22
	Cefepime (CPM30)	Gram-positive and Gram-negative	25	19-24	18
	Ceftriaxone (CRO30)	Gram-positive and Gram-negative	25	22-24	21
Tetracyclines	Tetracycline (TE30)	Gram-positive and Gram-negative	15	12-14	11
	Doxycycline (DO30)	Gram-positive and Gram-negative	16	13-15	12
Fluroquinolones	Ciprofloxacin (CIP5)	Gram-positive and Gram-negative	21	16-20	15
Macrolides	Azithromycin (AZM15)	Gram-positive and Gram-negative	18	14-17	13
	Erythromycin (E15)	Gram-positive and Gram-negative	23	14-22	13
Aminoglycosides	Amikacin (AK30)	Gram-positive and Gram-negative	17	15-16	14
	Gentamycin (CN10)	Gram-positive and Gram-negative	15	13-14	12
Sulfonamide	Trimethoprim-Sulfamethoxazole (SXT25)	Gram-positive and Gram-negative	16	11-15	10
Amphenicol	Chloramphenicol (C30)	Gram-positive and Gram-negative	18	13-17	12
Carbapenem	Meropenem (MEM10)	Gram-positive and Gram-negative	23	20-22	19

	Imipenem (IPM10)	Gram-positive and Gram-negative	23	20-22	19
Glycylcyline	Tigecycline (TGC15)	Gram-positive and Gram-negative	18	15-17	15
Glycopeptide	Vancomycin (VA30)	Gram-positive	25	22-24	21
Oxazolidinones	Linezolid (LZ30)	Gram-positive	26	23-25	22

S2 Table. List of all the Antibiotics Used in the Experiment.
