# Supplementary material for: Microbiological quality assessment of potential pathogenic bacteria and multidrug resistance patterns in commercial electrolyte drinks in Dhaka, Bangladesh
Source: PLoS One. 2026 Jun 2;21(6):e0336888. doi: 10.1371/journal.pone.0336888 (PMC13229343; doi:10.1371/journal.pone.0336888)
Supplement: S3 Table — The degree of sensitivity exhibited by the bacterial isolates to the antibiotics is shown. The number enclosed by the first bracket is the percentage of isolates. (PDF) [file pone.0336888.s003.pdf]

Supporting information

Antibiotics	Organism																							
	Klebsiella pneumoniae (N= 26)			*Escherichia coli* (N = 11)			*Pseudomonas aeruginosa* (N = 8)			*Acinetobacter baumannii* (N= 6)			*Vibrio spp.* (N = 10)			*Staphylococcus aureus* (N = 15)			*Staphylococcus epidermidis* (N= 16)			*Listeria spp.* (N = 18)		
	**R**	**I**	**S**	**R**	**I**	**S**	**R**	**I**	**S**	**R**	**I**	**S**	**R**	**I**	**S**	**R**	**I**	**S**	**R**	**I**	**S**	**R**	**I**	**S**
**Ampicillin**	-	-	-	8 (72.73)	1 (9.09)	2 (18.18)	-	-	-	-	-	-	4 (40.0)	4 (40.0)	2 (20.0)	10 (66.67)	2 (13.33)	3 (20.0)	12 (75.0)	3 (18.75)	1 (6.25)	15 (83.33)	2 (11.11)	1 (5.56)
**Amoxicillin**	-	-	-	9 (81.82)	0	3 (27.27)	-	-	-	-	-	-	6 (60.0)	2 (20.0)	2 (20.0)	12 (80.0)	1 (6.67)	2 (13.33)	13 (81.25)	1 (6.25)	2 (12.5)	14 (77.78)	1 (5.56)	3 (16.67)
**Amoxiclav**	16 (61.54)	6 (23.08)	6 (23.08)	6 (54.55)	3 (27.27)	2 (18.18)	-	-	-	-	-	-	2 (20.0)	3 (30.0)	5 (50.0)	4 (26.67)	6 (40.0)	5 (33.33)	11 (68.75)	5 (31.25)	0	5 (27.78)	9 (50.0)	4 (22.22)
**Cefoxitin**	6 (23.08)	10 (38.46)	10 (38.46)	5 (45.45)	3 (27.27)	3 (27.27)	4 (50.0)	1 (12.5)	3 (37.5)	3 (50.0)	2 (33.33)	1 (16.67)	3 (30.0)	3 (30.0)	4 (40.0)	4 (26.67)	5 (33.33)	6 (40.0)	9 (60.0)	0	7 (43.75)	6 (33.33)	7 (38.89)	5 (27.78)
**Cefotaxime**	16 (61.54)	4 (15.38)	6 (23.08)	4 (36.36)	4 (36.36)	3 (27.27)	-	-	-	5 (83.33)	1 (16.67)	0	3 (30.0)	3 (30.0)	4 (40.0)	11 (73.33)	3 (20.0)	1 (6.67)	4 (25.0)	7 (43.75)	5 (31.25)	9 (50.0)	4 (22.22)	5 (27.78)
**Cefepime**	14 (53.85)	7 (26.92)	5 (19.23)	8 (72.73)	0	3 (27.27)	3 (37.5)	1	4 (50.0)	4 (66.67)	0	2 (33.33)	2 (20.0)	6 (60.0)	2 (20.0)	9 (60.0)	2 (13.33)	4 (26.67)	7 (43.75)	7 (43.75)	2 (12.5)	6 (33.33)	4 (22.22)	8 (44.44)
**Ceftrixone**	20 (76.92)	2 (7.69)	4 (15.38)	5 (45.45)	4 (36.36)	2 (18.18)	-	-	-	2 (33.33)	3 (50.0)	1 (16.67)	2 (20.0)	6 (60.0)	2 (20.0)	11 (73.33)	4 (26.67)	0	13 (81.25)	3 (18.75)	0	7 (38.89)	5 (27.78)	6 (33.33)
**Tetracycline**	12 (46.15)	8 (30.77)	6 (23.08)	7 (63.64)	2 (18.18)	2 (18.18)	2 (25.0)	2 (25.0)	4 (50.0)	1 (16.67)	2 (33.33)	3 (50.0)	2 (20.0)	4 (40.0)	4 (40.0)	9 (60.0)	4 (26.67)	2 (13.33)	6 (37.5)	3 (18.75)	7 (43.75)	13 (72.22)	3 (16.67)	2 (12.5)
**Doxycycline**	11 (42.31)	8 (30.77)	7 (26.92)	6 (54.55)	1 (9.09)	4 (36.36)	3 (37.5)	1 (12.5)	4 (50.0)	2 (33.33)	3 (50.0)	1 (16.67)	5 (50.0)	2 (20.0)	3 (30.0)	7 (46.67)	3 (20.0)	5 (33.33)	4 (25.0)	7 (43.75)	5 (31.25)	4 (22.22)	8 (44.44)	6 (33.33)
**Ciprofloxacin**	12 (46.15)	6 (23.08)	8 (30.77)	3 (27.27)	4 (36.36)	4 (36.36)	5 (62.5)	2 (25.0)	1 (12.5)	4 (66.67)	1 (16.67)	1 (16.67)	0	5 (50.0)	5 (50.0)	6 (40.0)	5 (33.33)	4 (26.67)	2 (12.5)	8 (50.0)	6 (37.5)	12 (66.67)	1 (5.56)	5 (27.78)
**Azithromycin**	-	-	-	-	-	-	3 (37.5)	2 (25.0)	3 (37.5)	2 (33.33)	1 (16.67)	3 (50.0)	2 (20.0)	4 (40.0)	4 (40.0)	7 (46.67)	4 (26.67)	4 (26.67)	7 (43.75)	8 (50.0)	1 (6.25)	4 (22.22)	5 (27.78)	9 (50.0)
**Erythromycin**	-	-	-	-	-	-	3 (37.5)	0	5 (62.5)	3 (50.0)	1 (16.67)	2 (33.33)	3 (30.0)	4 (40.0)	3 (30.0)	9 (60.0)	4 (26.67)	2 (13.33)	9 (56.25)	4 (25.0)	3 (18.75)	6 (33.33)	9 (50.0)	3 (16.67)
**Amikacin**	9 (34.62)	10 (38.46)	7 (26.92)	2 (18.18)	3 (27.27)	6 (54.55)	1 (12.5)	1 (12.5)	6 (75.0)	4 (66.67)	0	2 (33.33)	2 (20.0)	3 (30.0)	5 (50.0)	3 (20.0)	7 (46.67)	5 (33.33)	8 (50.0)	3 (18.75)	5 (31.25)	5 (27.78)	7 (38.89)	6 (33.33)
**Gentamicin**	8 (30.77)	7 (26.92)	11 (42.31)	3 (27.27)	5 (45.45)	3 (27.27)	2 (25.0)	4 (50.0)	2 (25.0)	5 (83.33)	0	1 (16.67)	1 (10.0)	3 (30.0)	6 (60.0)	5 (33.33)	3 (20.0)	7 (46.67)	9 (60.0)	3 (18.75)	4 (25.0)	3 (16.67)	10 (55.56)	5 (27.78)
**Trimethoprim-Sulfamethoxazole**	15 (57.69)	6 (23.08)	5 (19.23)	9 (81.82)	2 (18.18)	0	4 (50.0)	2 (25.0)	2 (25.0)	4 (66.67)	2 (33.33)	0	1 (10.0)	4 (40.0)	5 (50.0)	4 (26.67)	7 (46.67)	4 (26.67)	4 (25.0)	5 (31.25)	7 (43.75)	2 (11.11)	7 (38.89)	9 (50.0)
**Chloramphenicol**	9 (34.62)	8 (30.77)	9 (34.62)	5 (45.45)	1 (9.09)	5 (45.45)	-	-	-	-	-	-	2 (20.0)	4 (40.0)	4 (40.0)	7 (46.67)	5 (33.33)	3 (20.0)	3 (18.75)	4 (25.0)	9 (56.25)	6 (33.33)	4 (22.22)	8 (44.44)
**Meropenem**	10 (38.46)	6 (23.08)	10 (38.46)	2 (18.18)	4 (36.36)	5 (45.45)	3 (37.5)	4 (50.0)	1 (12.5)	2 (33.33)	1 (16.67)	3 (50.0)	4 (40.0)	1 (10.0)	5 (50.0)	10 (66.67)	4 (26.67)	1 (6.67)	2 (12.5)	5 (31.25)	9 (56.25)	5 (27.78)	3 (16.67)	10 (55.56)
**Imipenem**	7 (26.92)	7 (26.92)	12 (46.15)	1 (9.09)	4 (36.36)	6 (54.55)	4 (50.0)	3 (37.5)	1 (12.5)	3 (50.0)	2 (33.33)	1 (16.67)	2 (20.0)	3 (30.0)	5 (50.0)	2 (13.33)	6 (40.0)	7 (46.67)	4 (25.0)	8 (50.0)	4 (25.0)	4 (22.22)	2 (11.11)	12 (66.67)
**Tigecycline**	4 (15.38)	8 (30.77)	14 (53.85)	2 (18.18)	3 (27.27)	6 (54.44)	3 (37.5)	1 (12.5)	4 (50.0)	1 (16.67)	1 (16.67)	4 (66.67)	2 (20.0)	3 (30.0)	5 (50.0)	1 (6.67)	3 (20.0)	11 (73.33)	2 (12.5)	6 (37.5)	8 (50.0)	4 (22.22)	9 (50.0)	5 (27.78)
**Vancomycin**	-	-	-	-	-	-	-	-	-	-	-	-	-	-	-	2 (13.33)	4 (26.67)	9 (60)	3 (18.75)	4 (25.0)	9 (56.75)	2 (11.11)	4 (22.22)	12 (66.67)
**Linezolid**	-	-	-	-	-	-	-	-	-	-	-	-	-	-	-	3 (20.0)	2 (13.33)	10 (66.67)	2 (12.5)	2 (12.5)	12 (75.0)	3 (16.67)	4 (22.22)	11 (61.11)

S3 Table. Sensitivity profile of all isolated organisms.
