# Supplementary figures and images for: Microbiological quality assessment of potential pathogenic bacteria and multidrug resistance patterns in commercial electrolyte drinks in Dhaka, Bangladesh
Source: PLoS One. 2026 Jun 2;21(6):e0336888. doi: 10.1371/journal.pone.0336888 (PMC13229343; doi:10.1371/journal.pone.0336888)

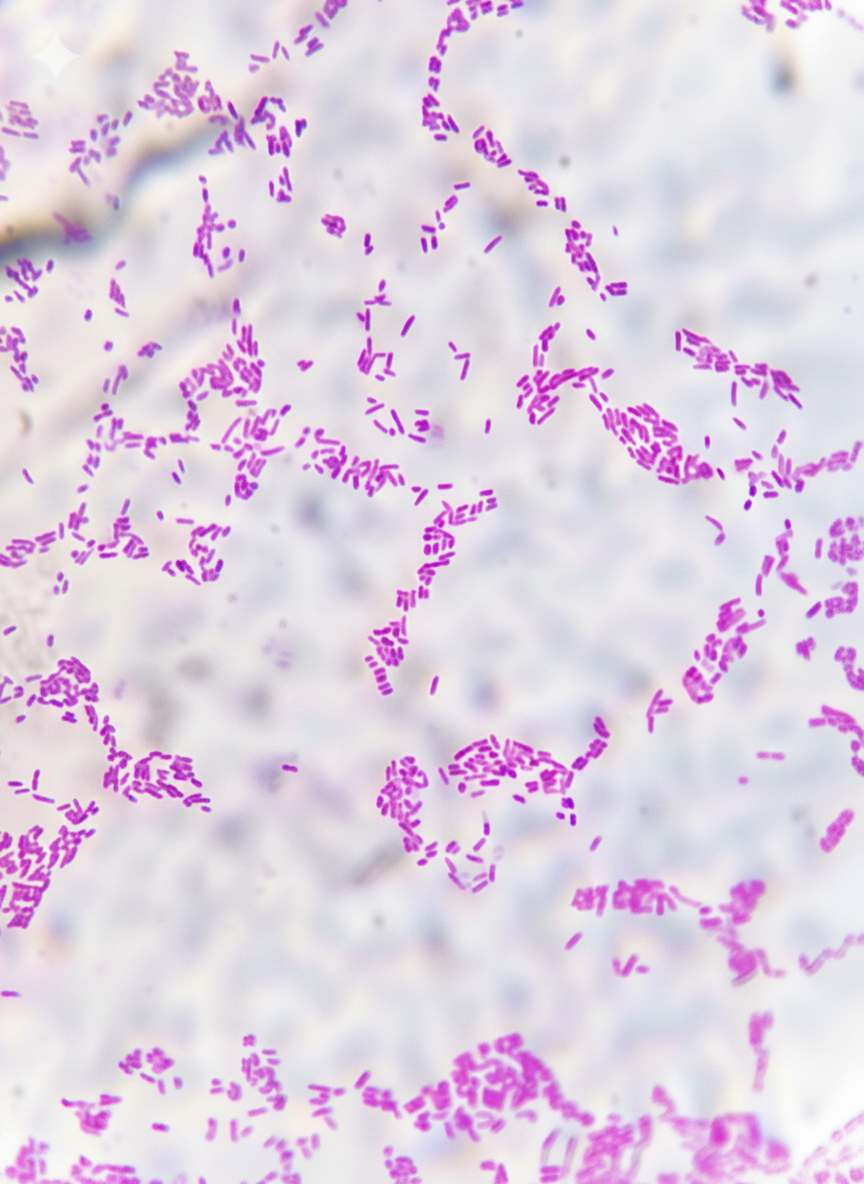

Supplement: S1 Fig — Here, the bacterial cells are appearing as Gram-negative, short rod-shaped bacilli appearing pink under light microscopy. (TIF) [file pone.0336888.s004.tif]

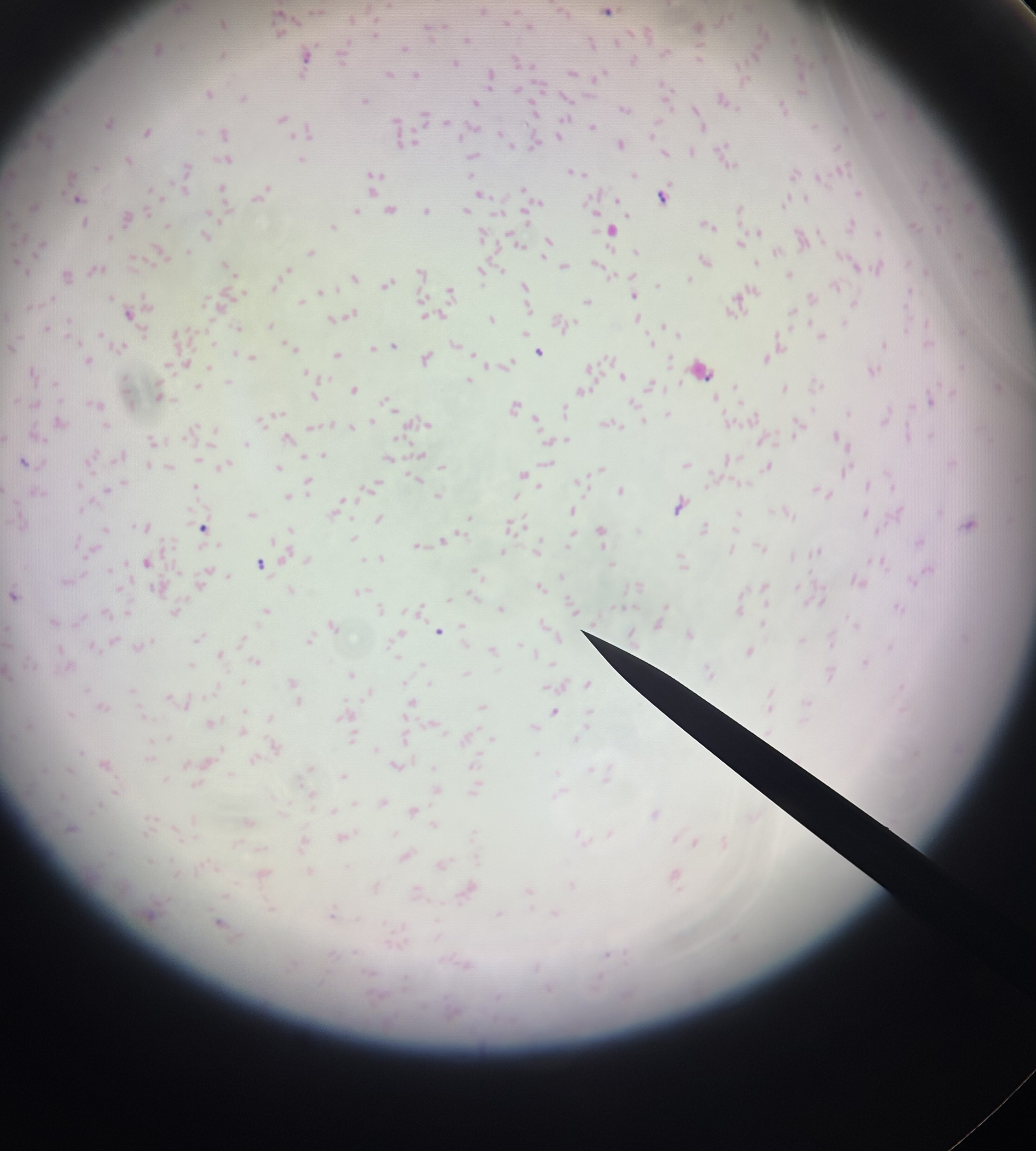

Supplement: S2 Fig — Here, the bacterial cells are appearing as Gram-negative, short rod-shaped bacteria appearing pink under light microscopy. (TIF) [file pone.0336888.s005.tif]

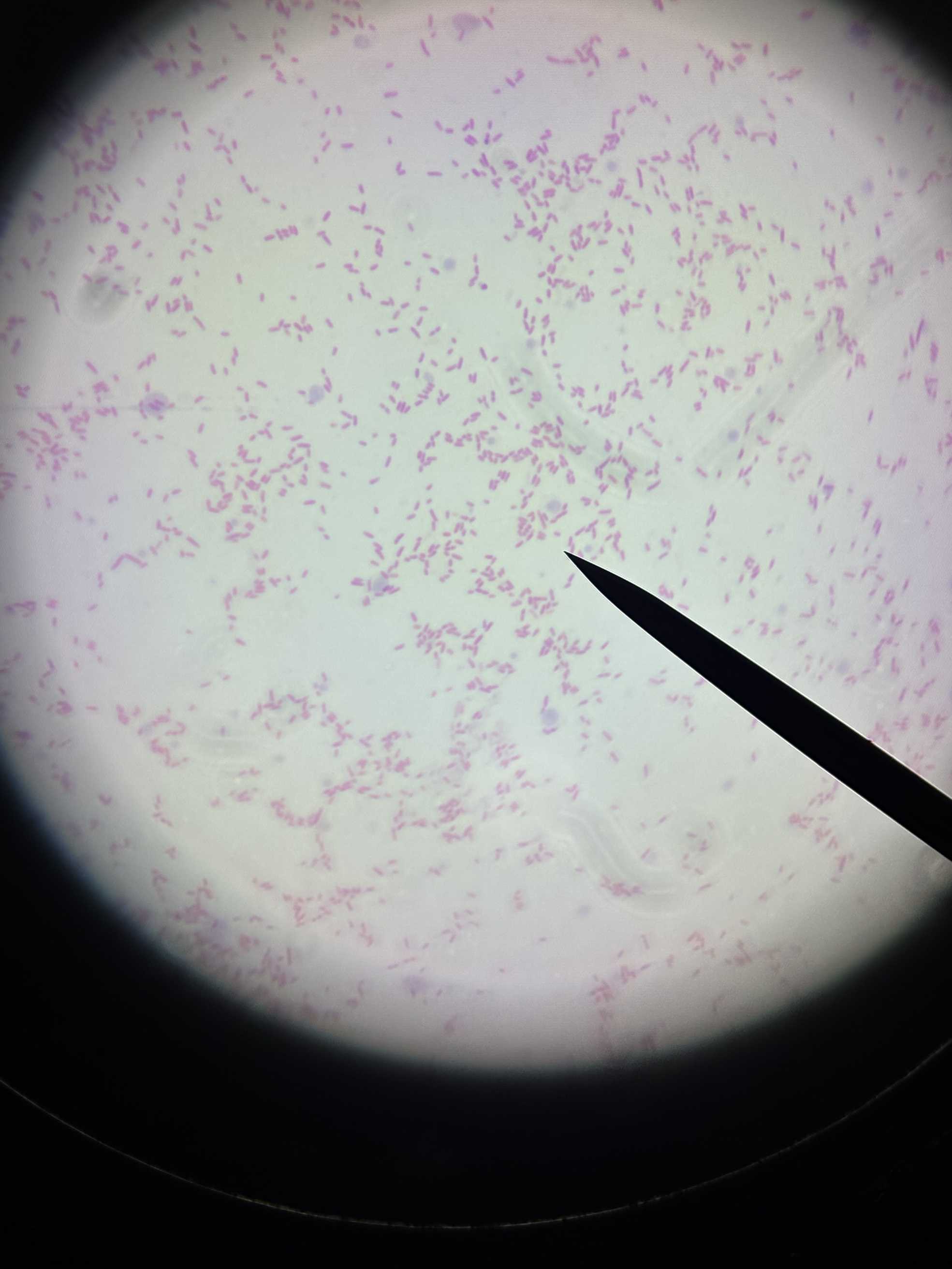

Supplement: S3 Fig — Here, the bacterial cells are appearing Gram-negative, rod-shaped cells appearing pink under light microscopy. (TIF) [file pone.0336888.s006.tif]

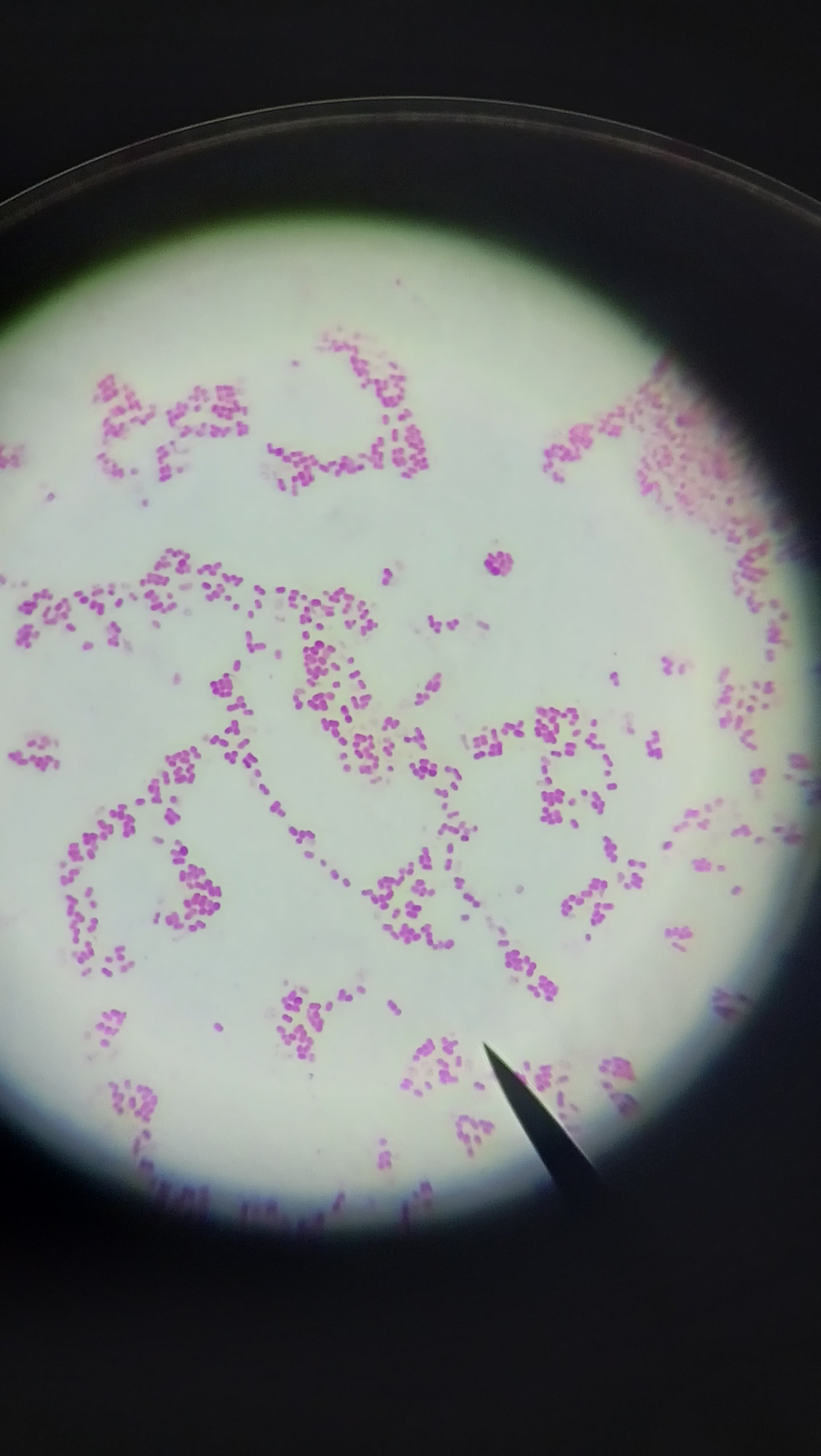

Supplement: S4 Fig — Here, the bacterial cells are appearing Gram-negative coccobacilli appearing as short rods, stained pink under light microscopy. (TIF) [file pone.0336888.s007.tif]

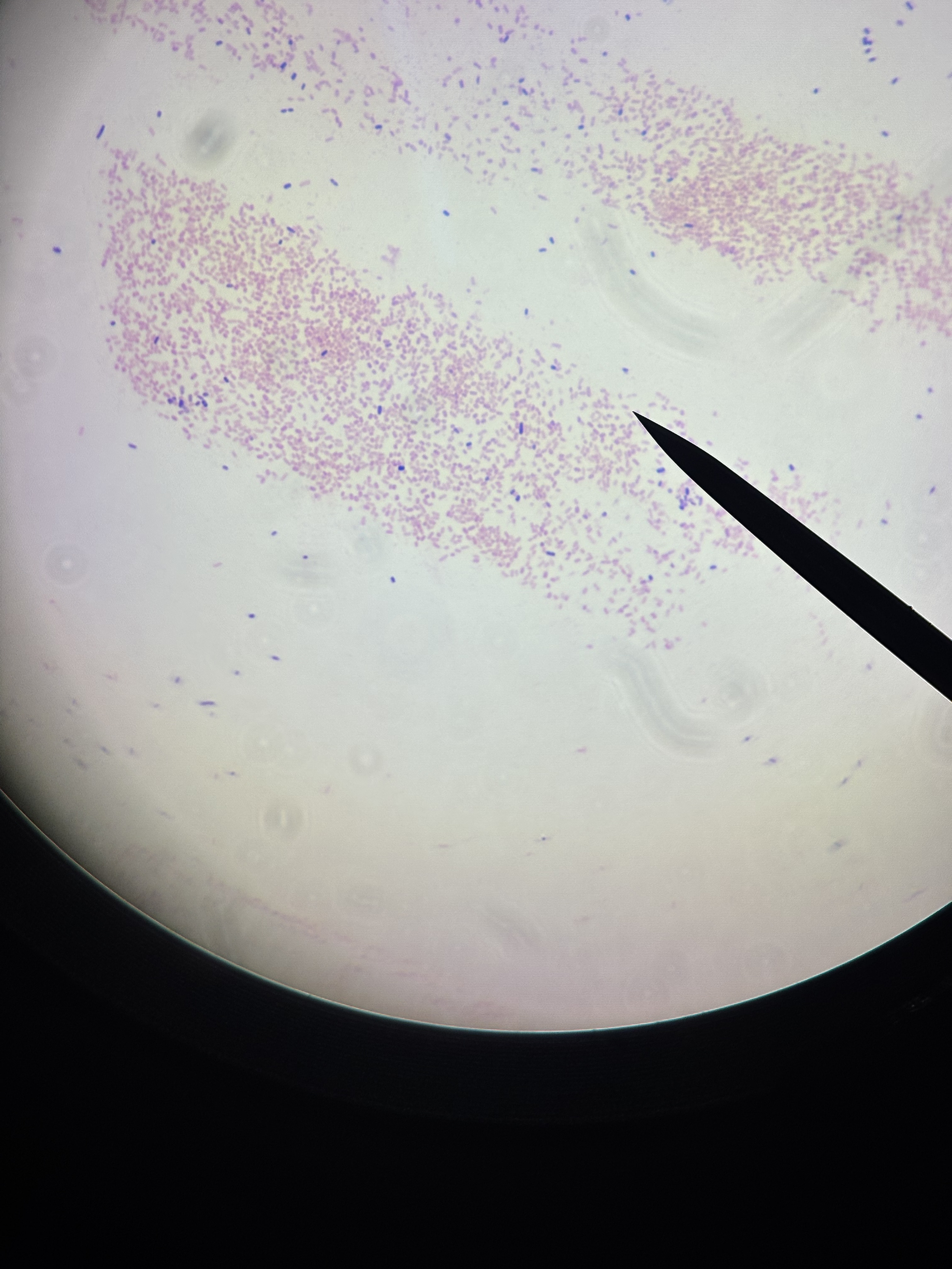

Supplement: S5 Fig — Here, the bacterial cells are appearing Gram-negative, curved rod-shaped cells under light microscopy. (TIF) [file pone.0336888.s008.tif]

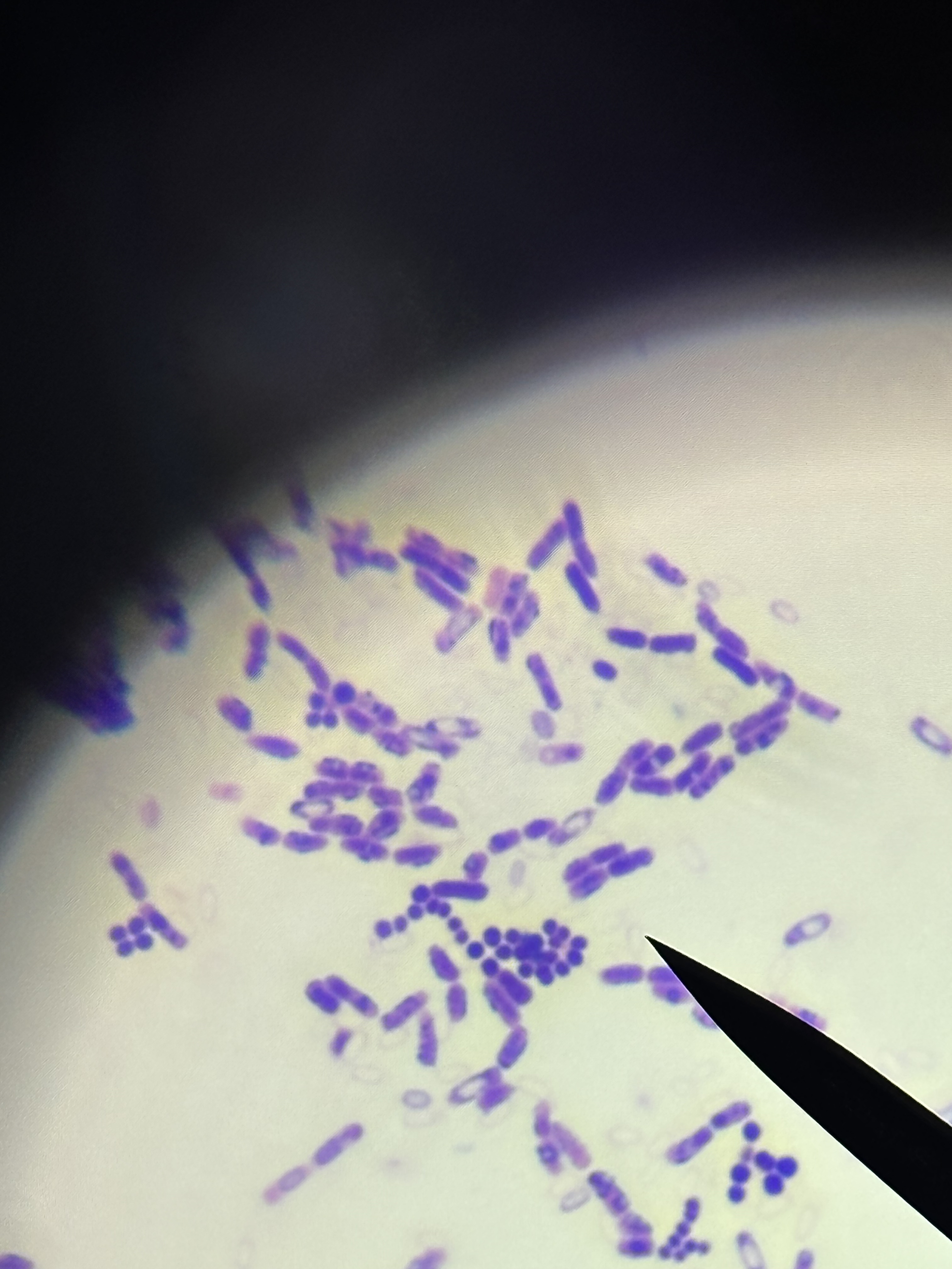

Supplement: S6 Fig — Here, the bacterial cells are appearing purple-stained Gram-positive cocci arranged in clusters, under light microscopy. (TIF) [file pone.0336888.s009.tif]

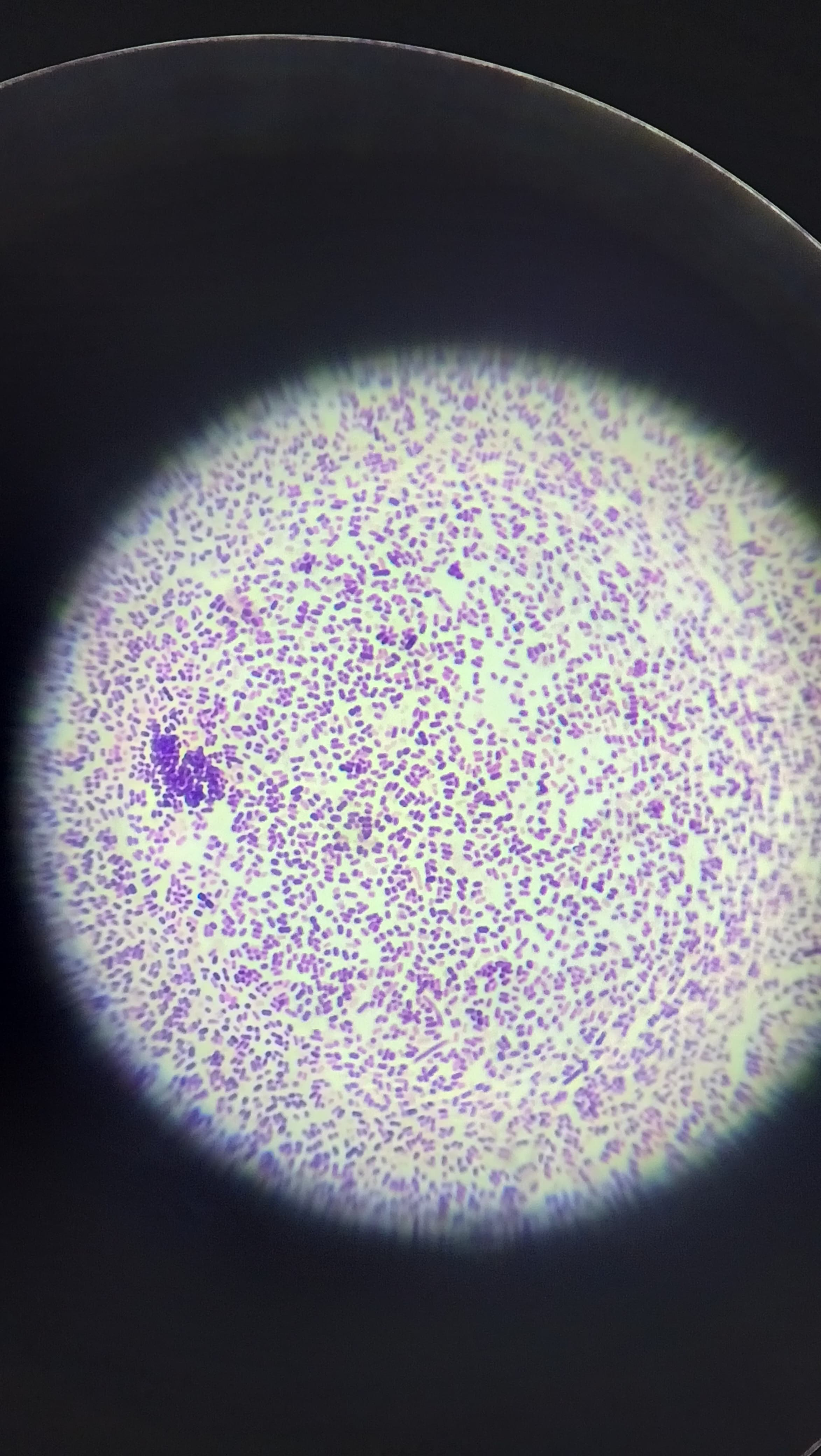

Supplement: S7 Fig — Here, the bacterial cells are appearing Gram-positive cocci appearing as grape-like clusters under light microscopy. (TIF) [file pone.0336888.s010.tif]

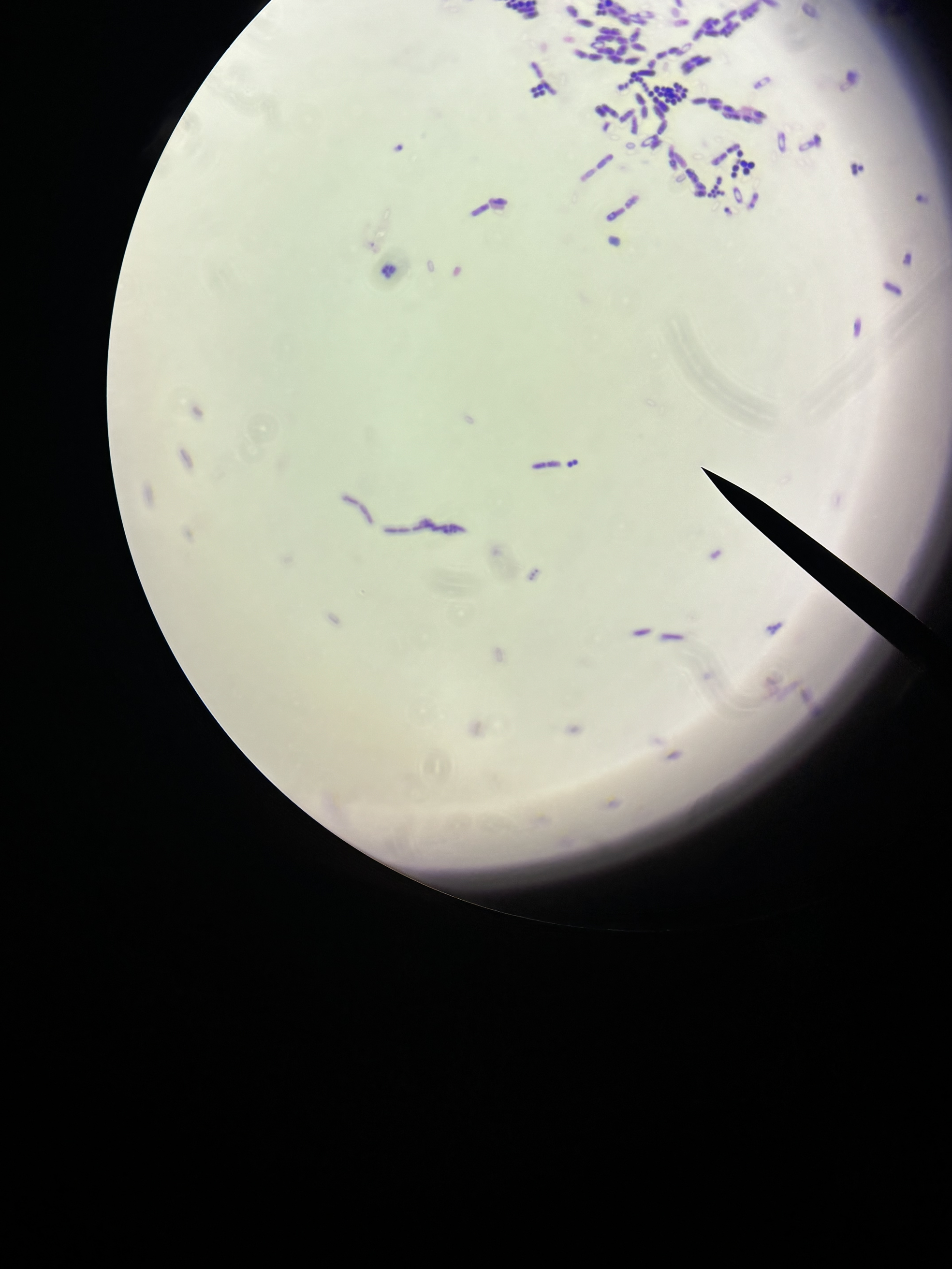

Supplement: S8 Fig — Here, the bacterial cells are appearing purple-stained Gram-positive, short rod-shaped bacteria under light microscopy. (TIF) [file pone.0336888.s011.tif]

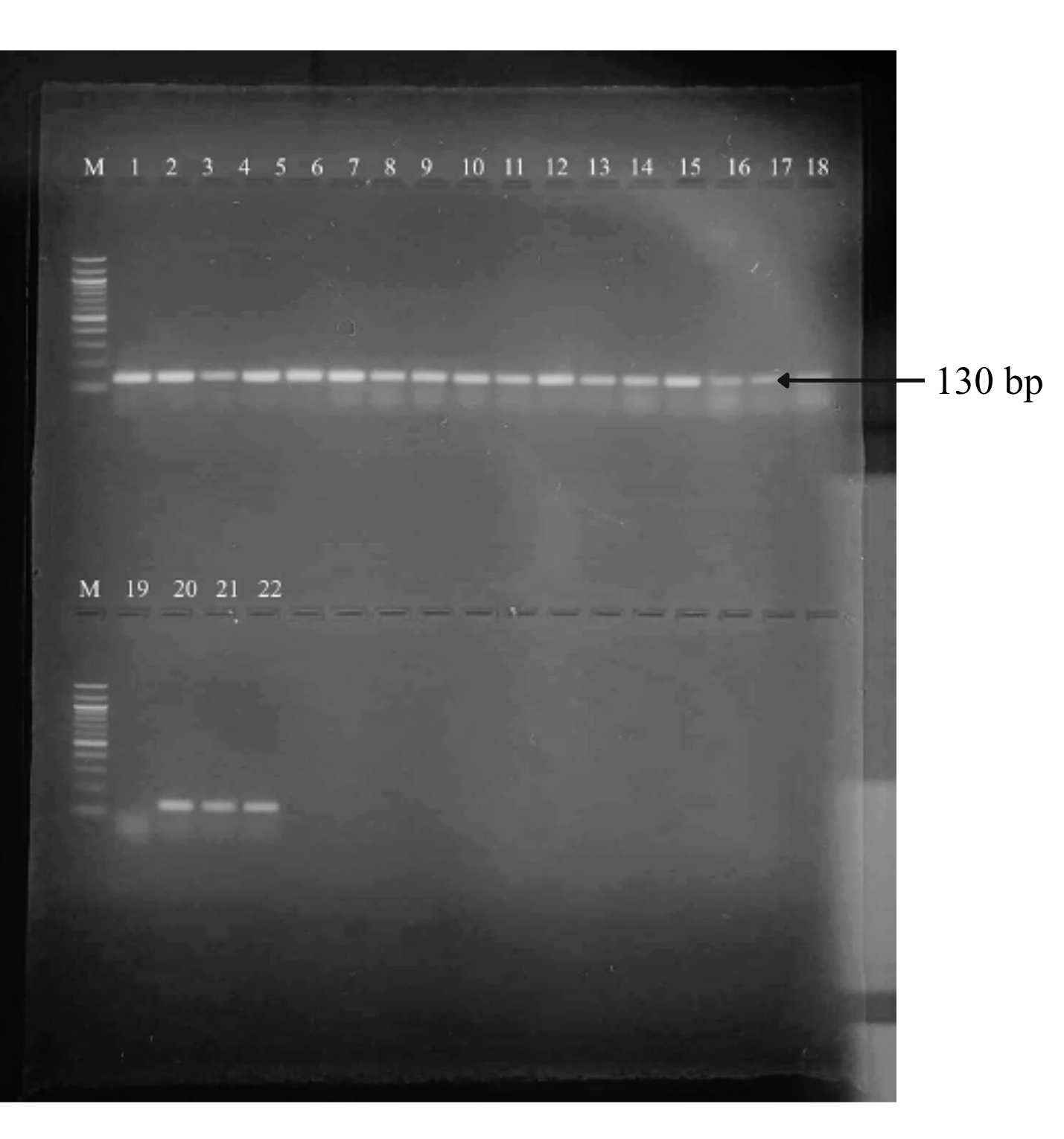

Supplement: S9 Fig — Here Lane (M) is a 100 bp DNA marker, and Lane (1–18, 20–22) are some positive samples at 130 bp. (TIF) [file pone.0336888.s012.tif]

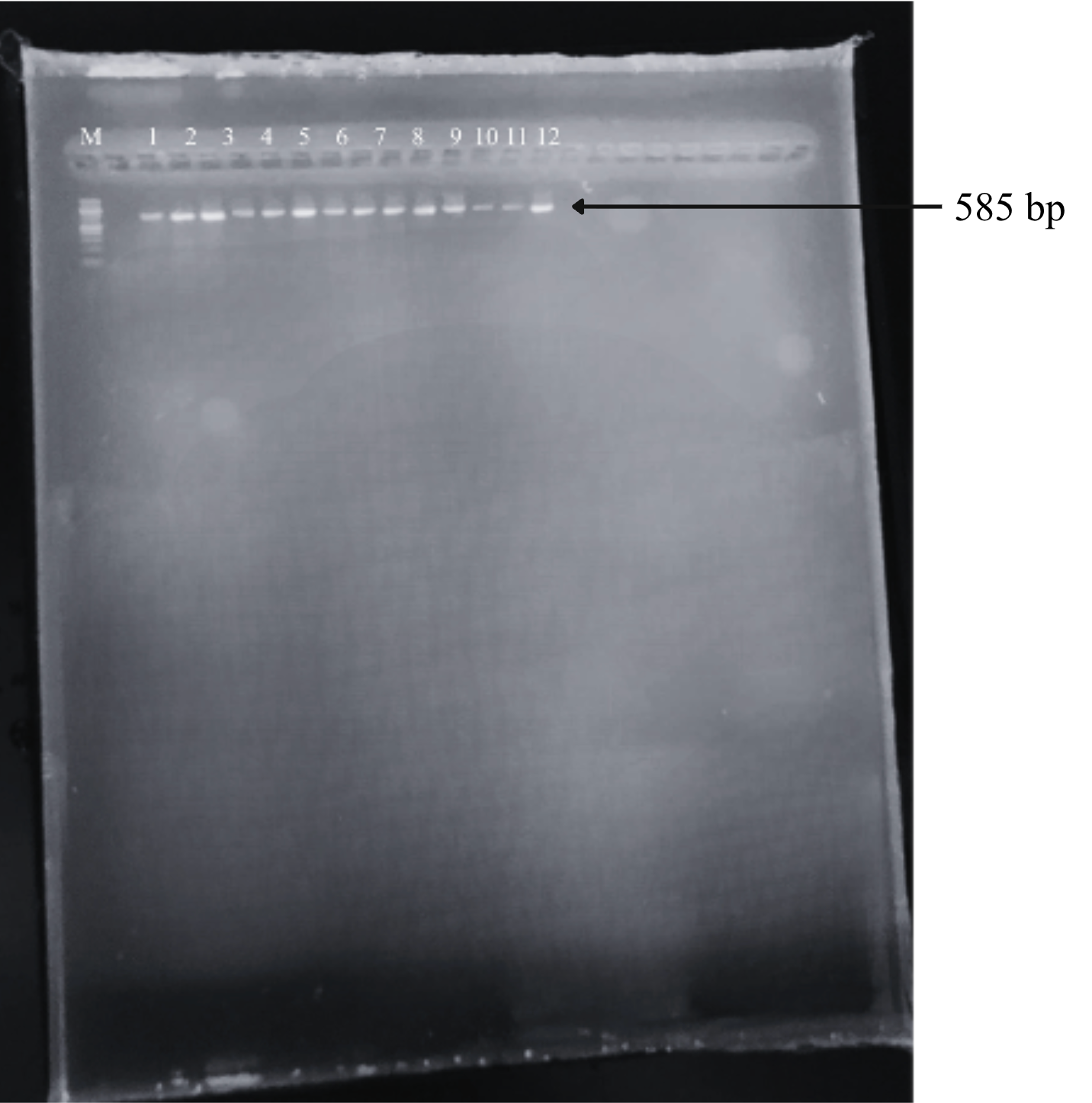

Supplement: S10 Fig — Here Lane (M) is a 100 bp DNA marker, and Lane (1–9) are some positive samples at 585 bp. (TIF) [file pone.0336888.s013.tif]

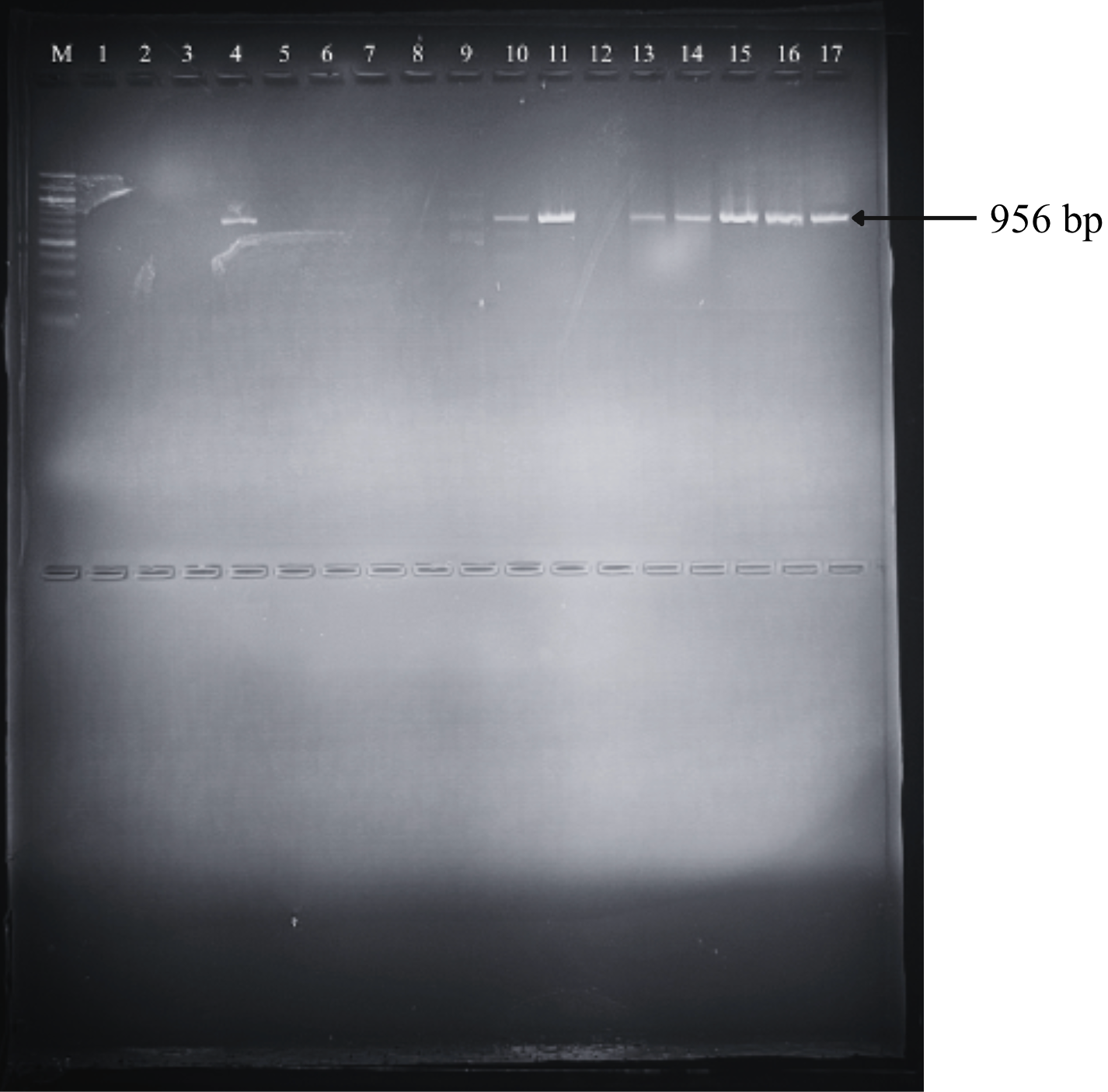

Supplement: S11 Fig — Here Lane (M) is a 100 bp DNA marker, and Lane (4, 10, 11, 13–17) are some positive samples at 956 bp. (TIF) [file pone.0336888.s014.tif]

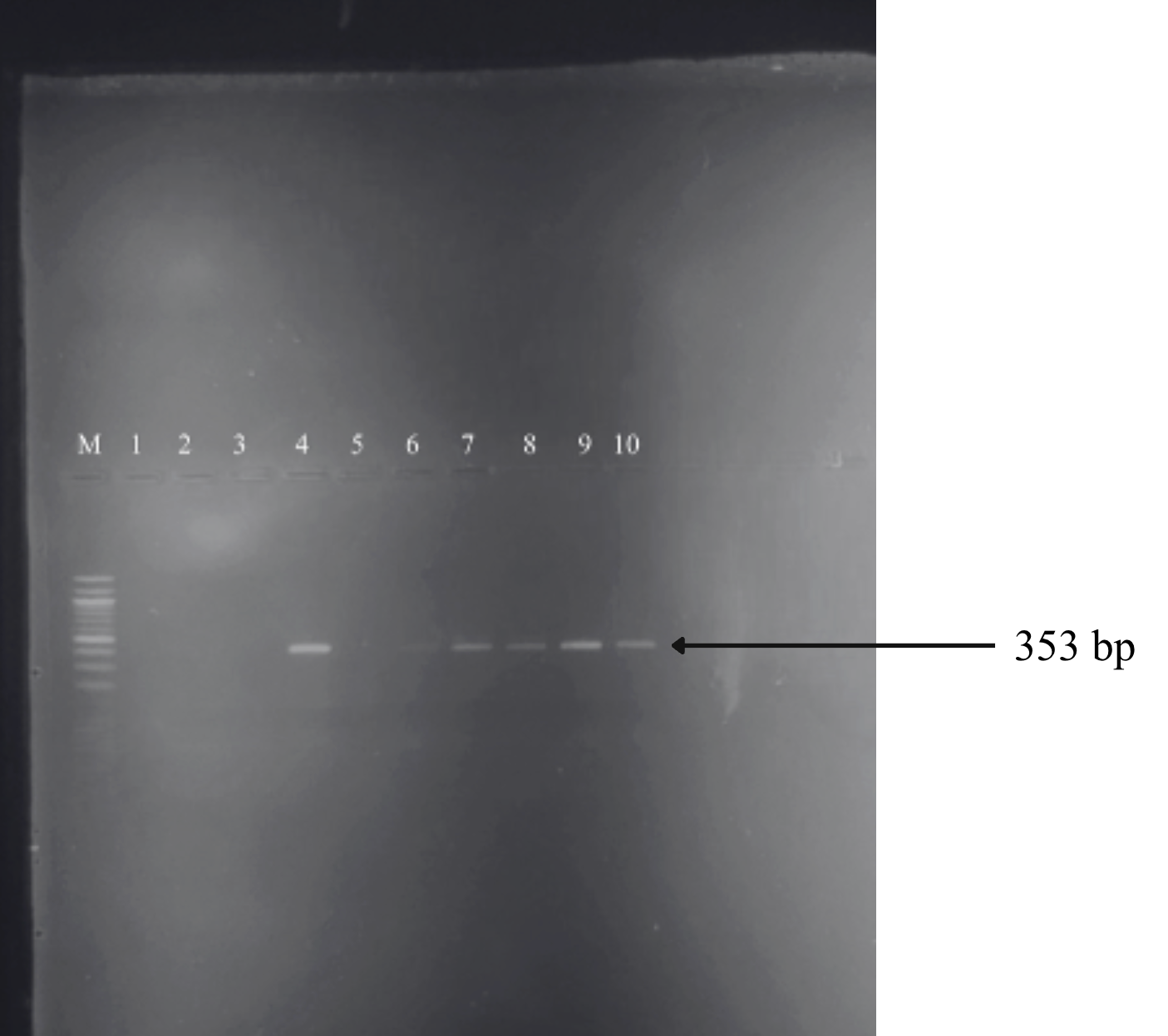

Supplement: S12 Fig — Here Lane (M) is a 100 bp DNA marker, and Lane (4, 6–10) are some positive samples at 353 bp. (TIF) [file pone.0336888.s015.tif]

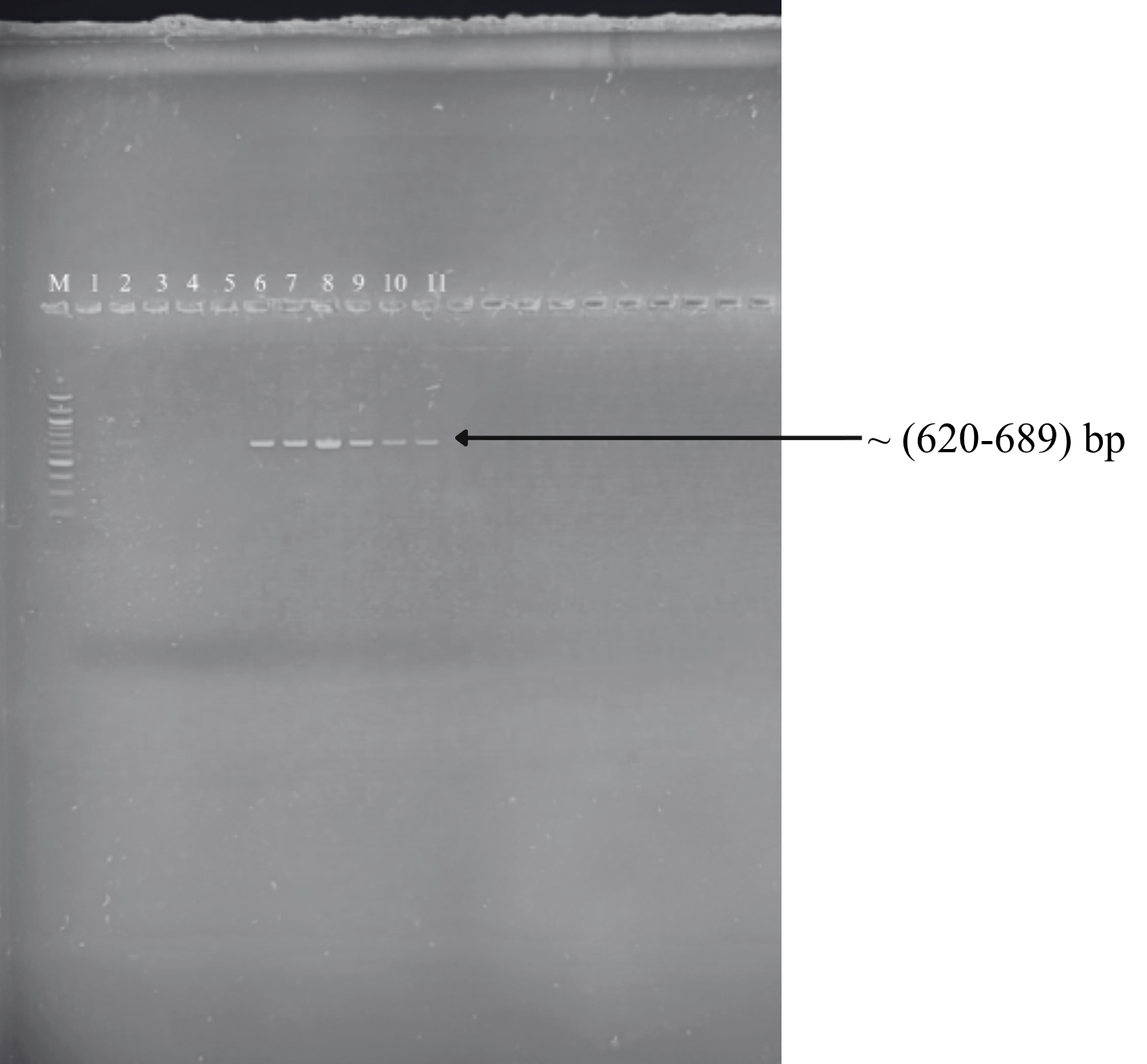

Supplement: S13 Fig — Here Lane (M) is a 100 bp DNA marker, and Lane (6–11) are some positive samples at ~ (620–689) bp. (TIF) [file pone.0336888.s016.tif]

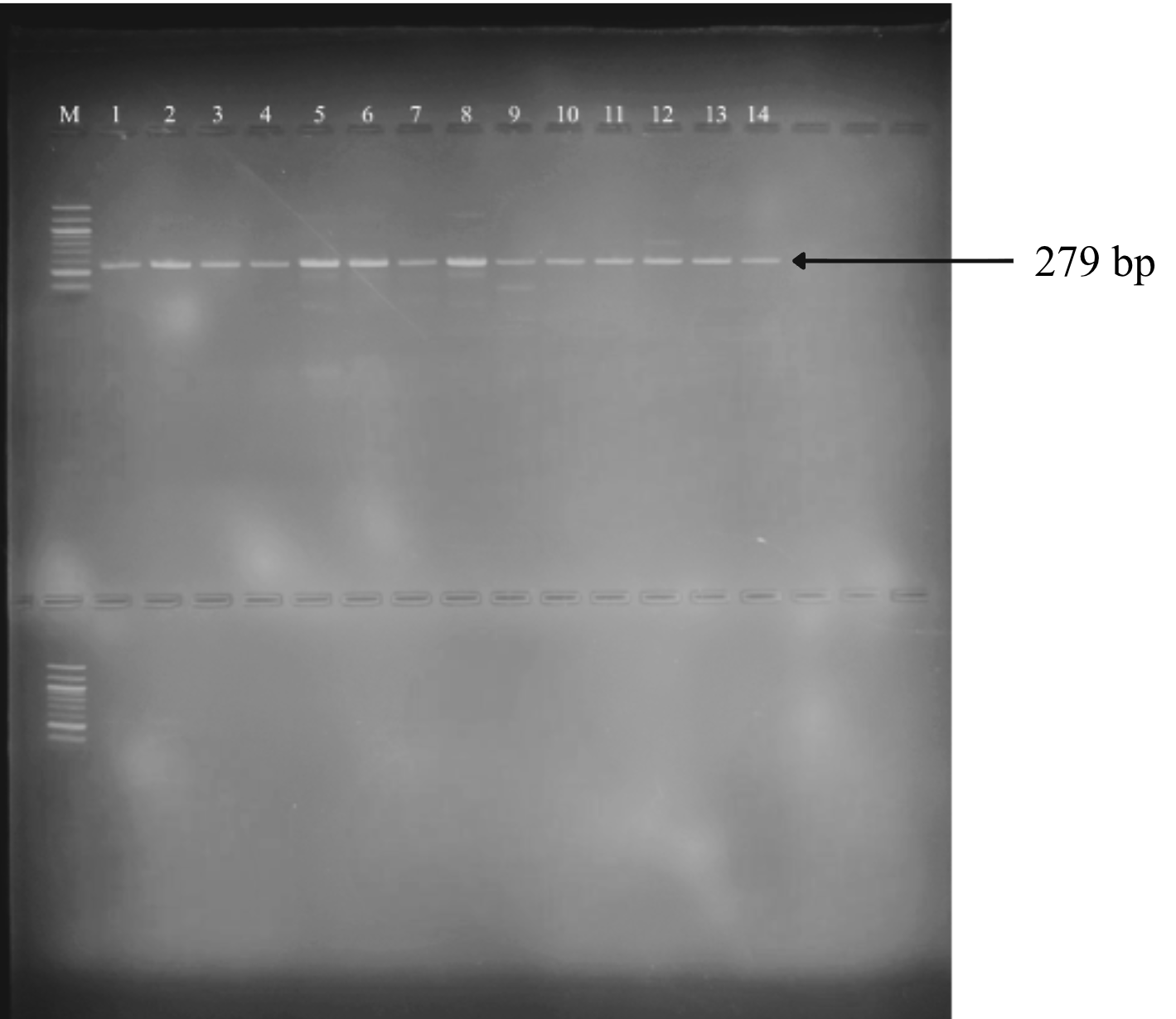

Supplement: S14 Fig — Here Lane (M) is a 100 bp DNA marker, and Lane (1–14) are some positive samples at 279 bp. (TIF) [file pone.0336888.s017.tif]

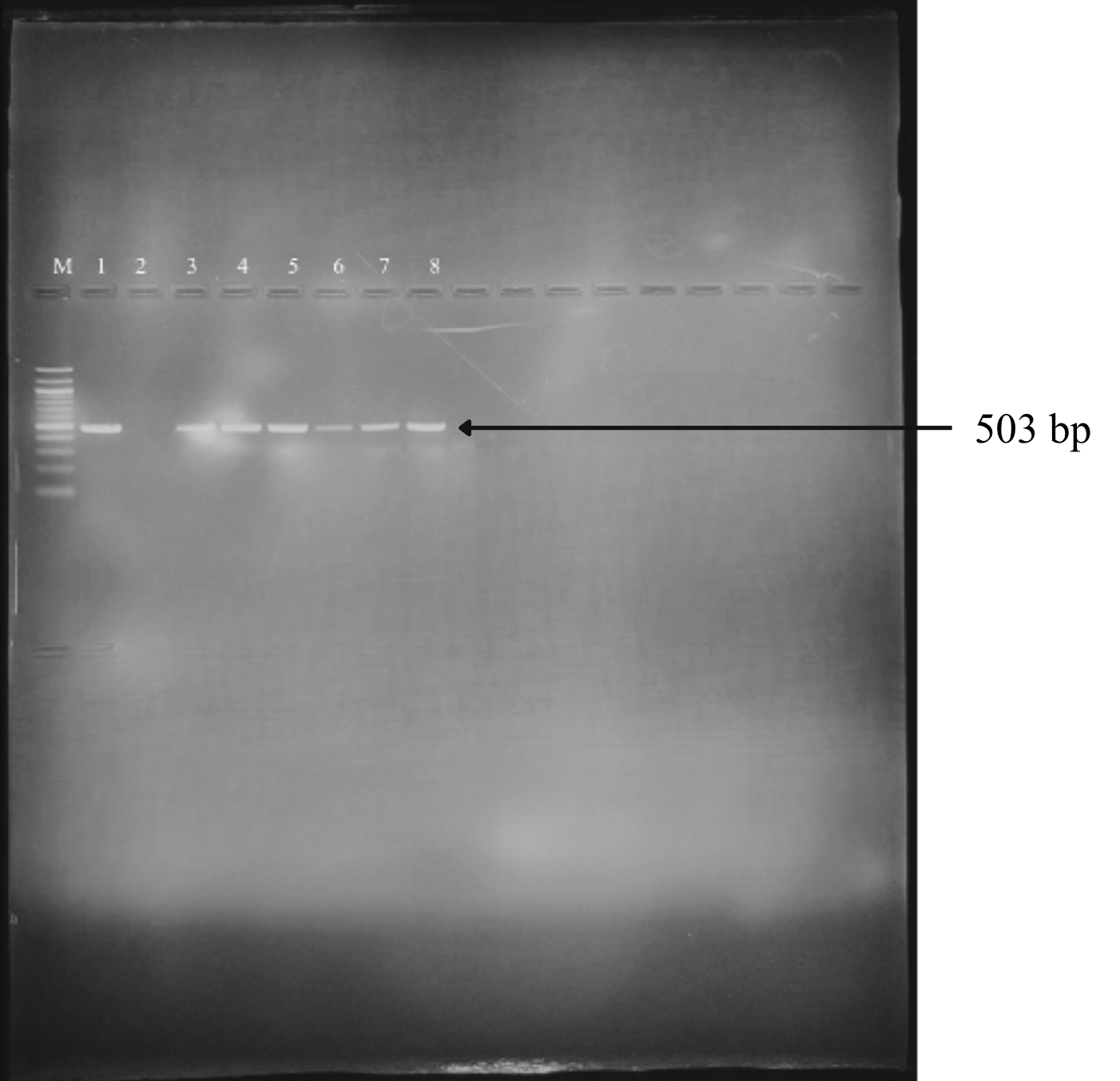

Supplement: S15 Fig — Here Lane (M) is a 100 bp DNA marker, and Lane (1, 3–8) are some positive samples at 503 bp. (TIF) [file pone.0336888.s018.tif]

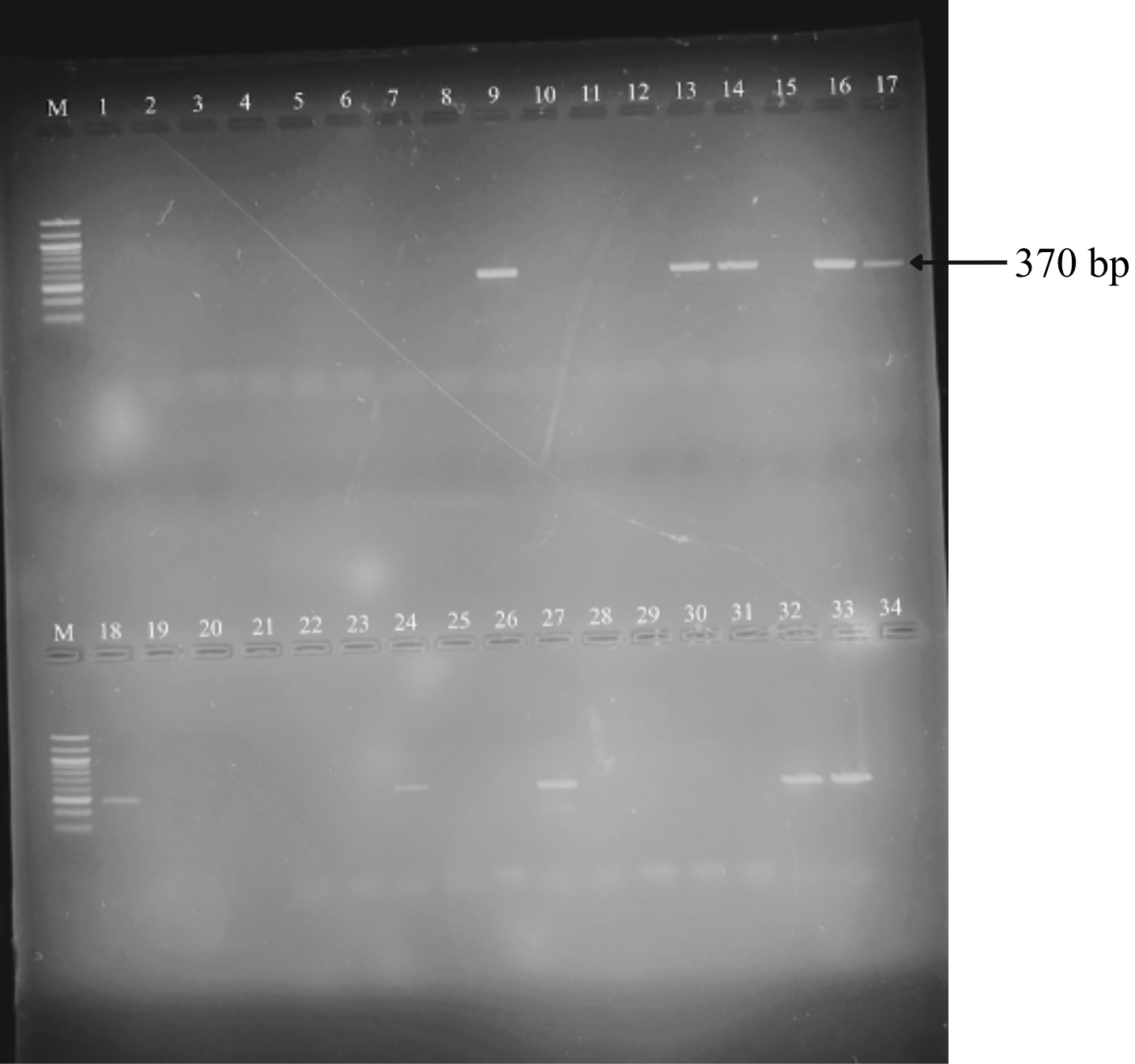

Supplement: S16 Fig — Here Lane (M) is a 100 bp DNA marker, and Lane (9, 13, 14, 16–18, 24, 27, 32–33) are some positive samples at 370 bp. (TIF) [file pone.0336888.s019.tif]

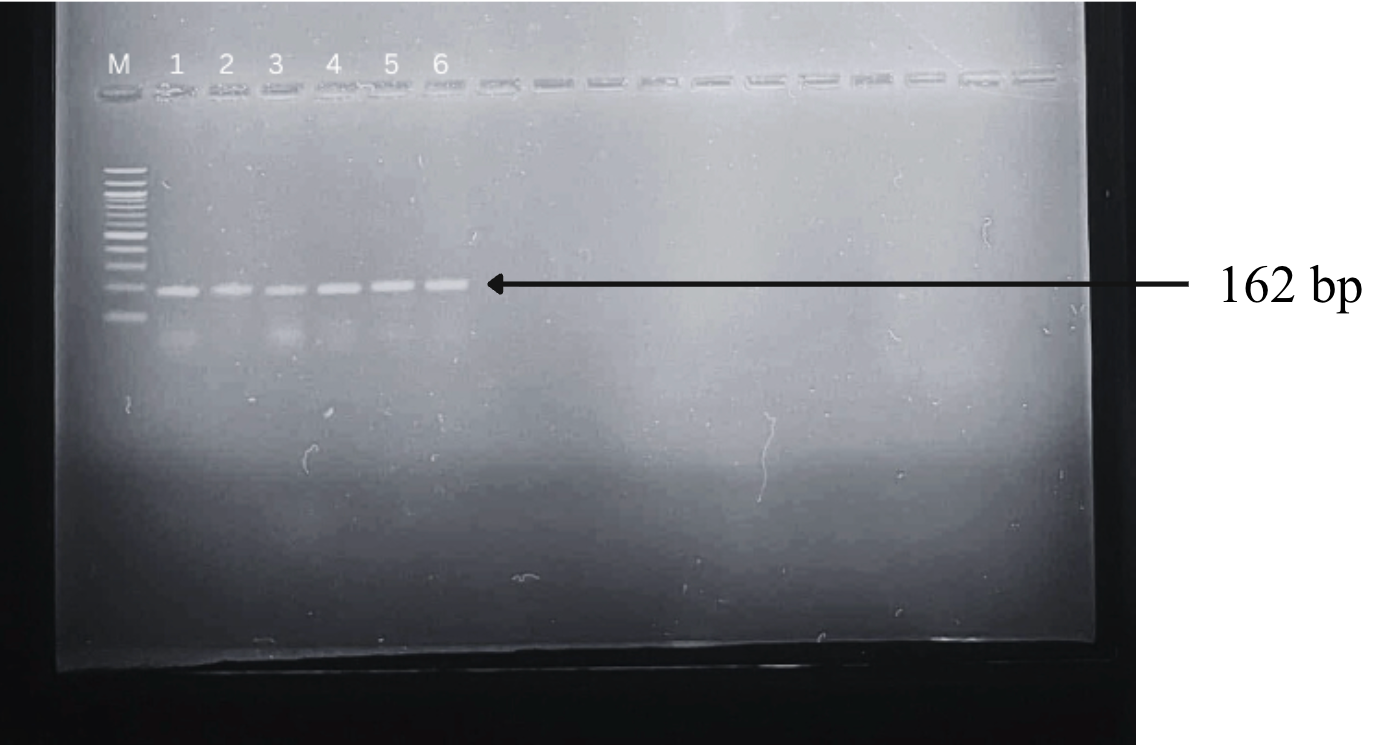

Supplement: S17 Fig — Here, Lane (M) is a 100 bp DNA marker, and Lane (1–6) are some positive samples at 162 bp. (TIF) [file pone.0336888.s020.tif]

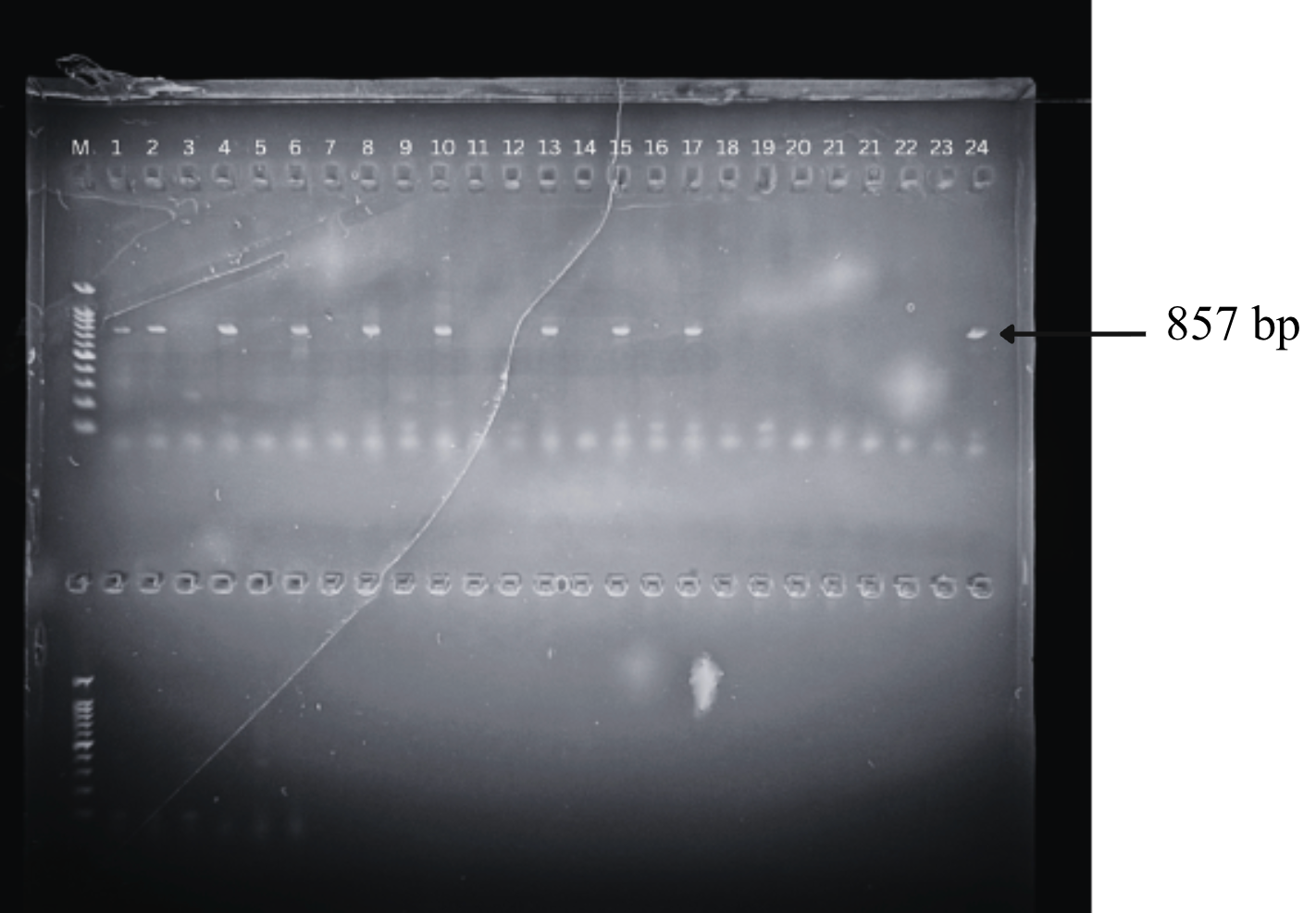

Supplement: S18 Fig — Here, Lane (M) is a 100 bp DNA marker, and Lane (1, 2, 4, 6, 8, 10, 13, 15, 17, 24) are some positive samples at 857 bp. (TIF) [file pone.0336888.s021.tif]

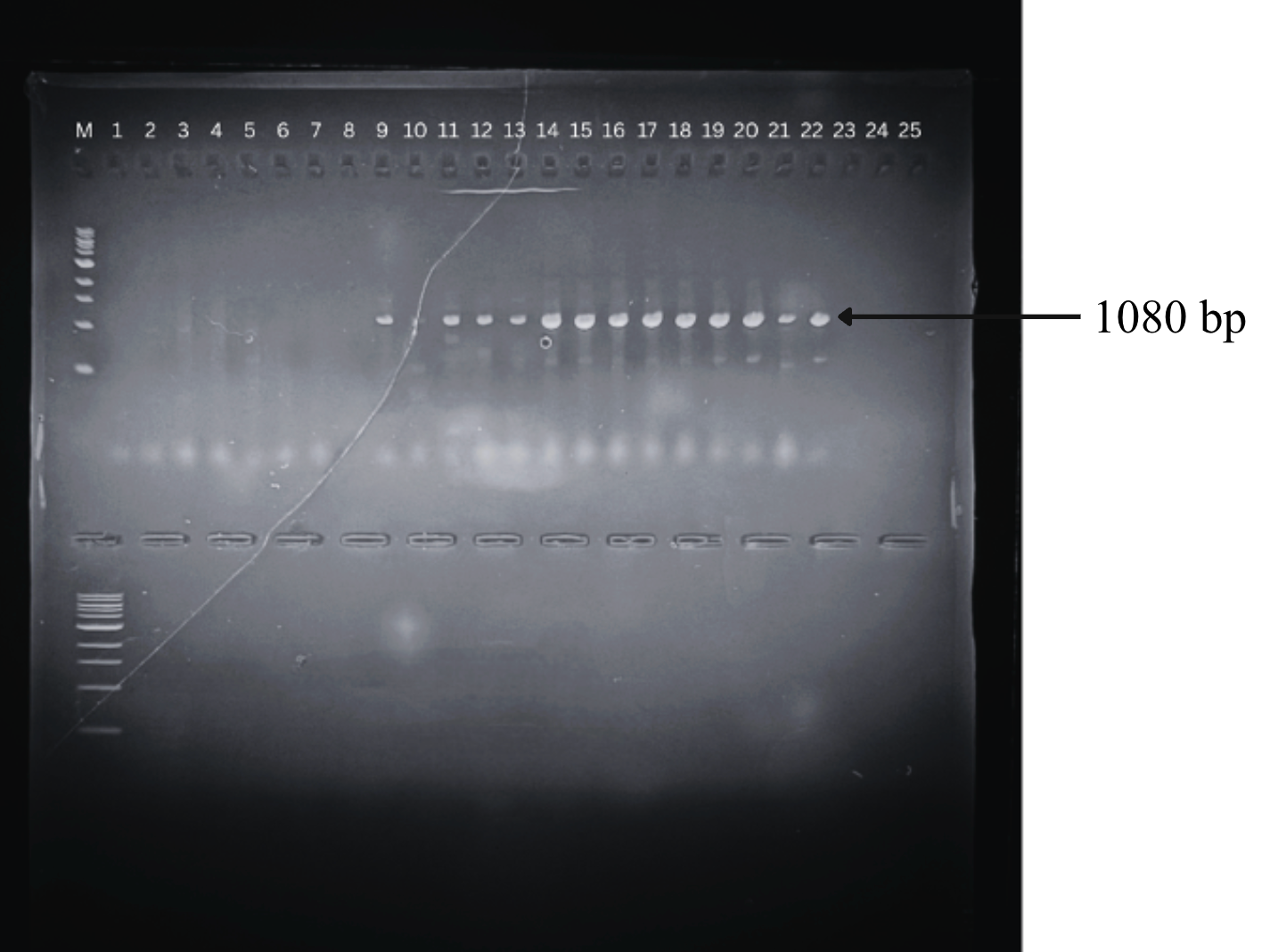

Supplement: S19 Fig — Here, Lane (M) is a 1k bp DNA marker, and Lane (9–22) are some positive samples at 1080 bp. (TIF) [file pone.0336888.s022.tif]

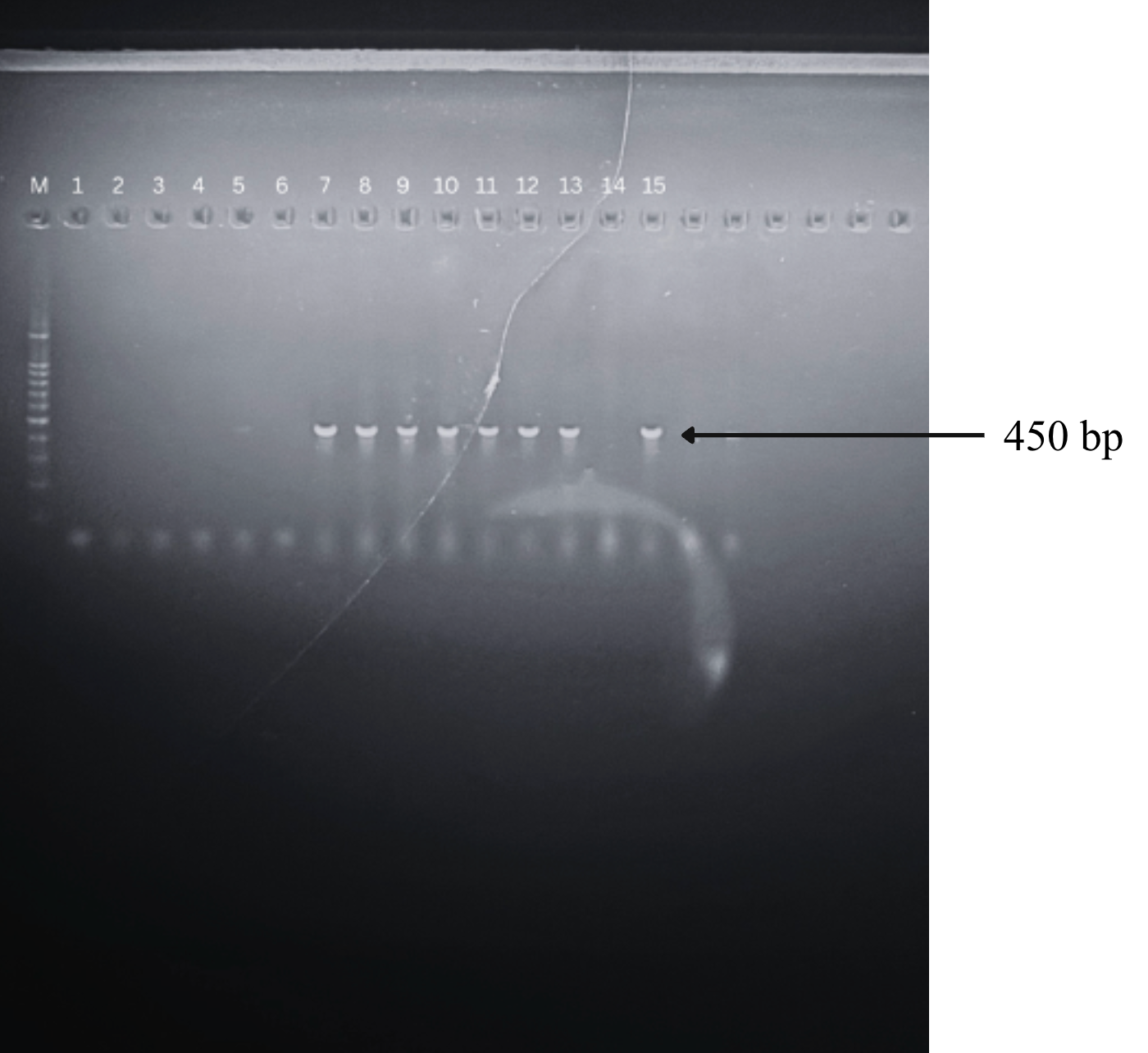

Supplement: S20 Fig — Here, Lane (M) is a 100 bp DNA marker and Lane (7–13, 15) are some positive samples at 450 bp. (TIF) [file pone.0336888.s023.tif]

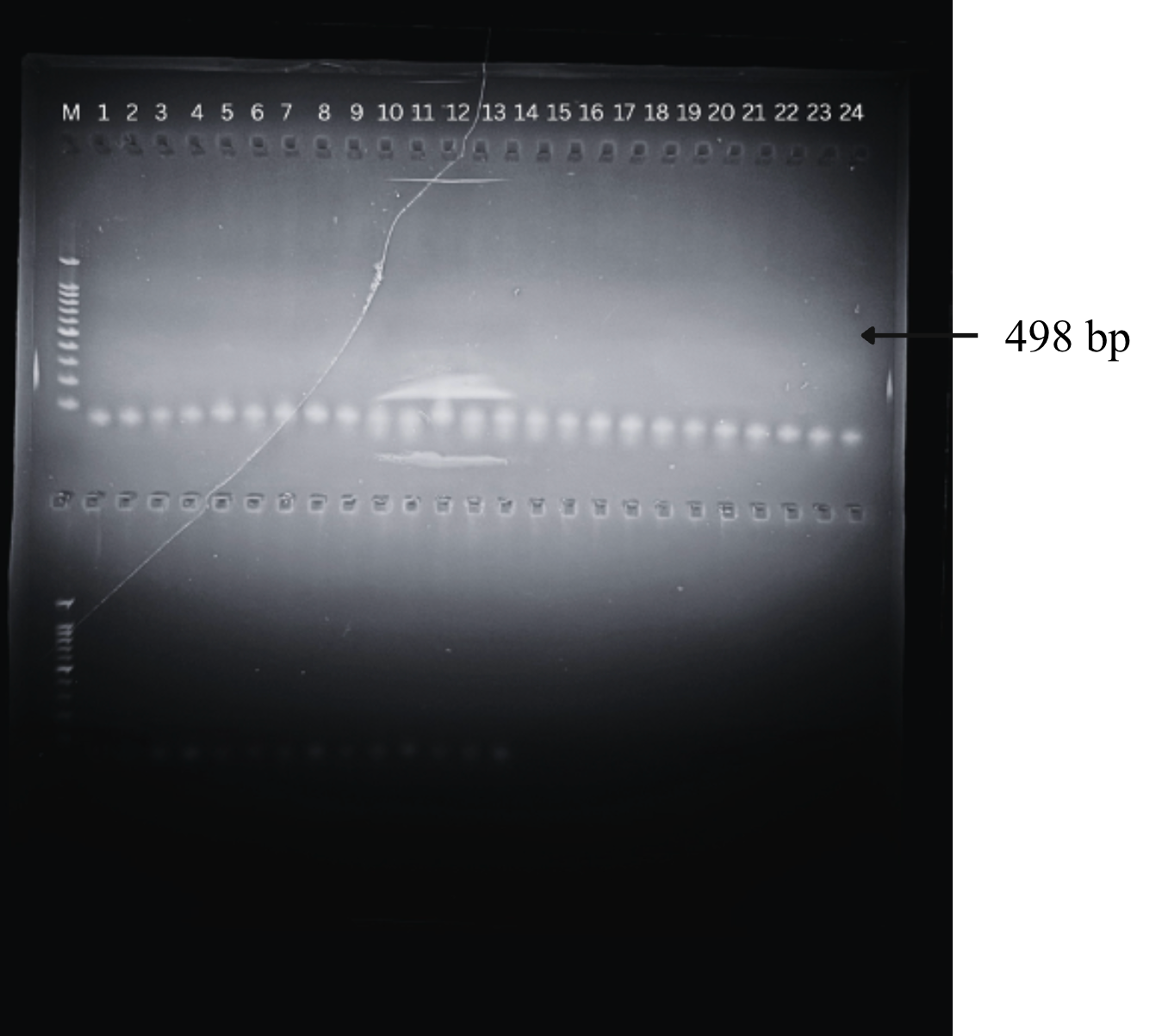

Supplement: S21 Fig — Here, Lame (M) is a 100 bp DNA marker, and Lane (1–24) are some negative samples that did not give any band at 498 bp. (TIF) [file pone.0336888.s024.tif]

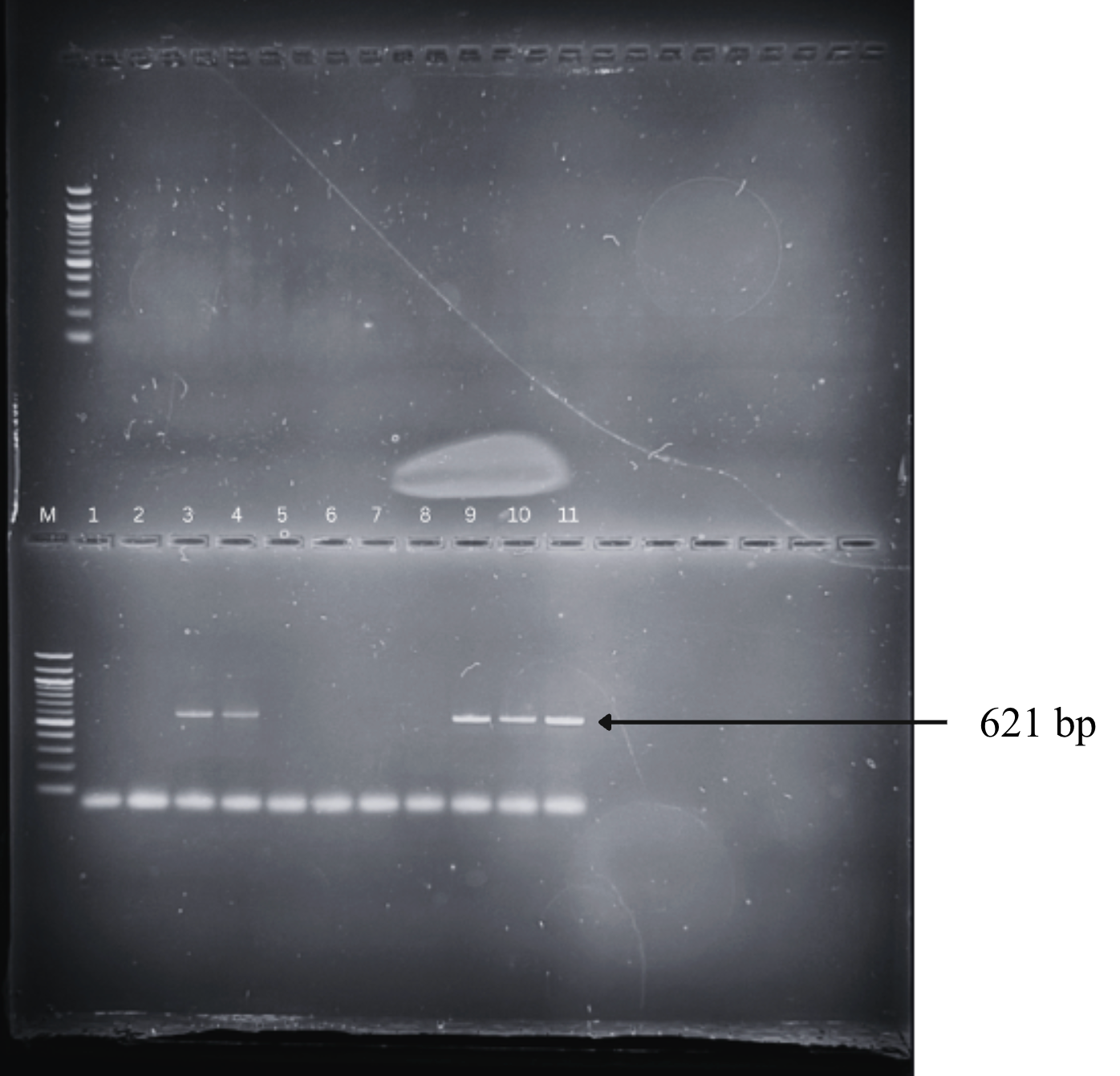

Supplement: S22 Fig — Here, Lane (M) is a 100 bp DNA marker and Lane (3, 4, 9, 10, 11) are some positive samples at 621 bp. (TIF) [file pone.0336888.s025.tif]

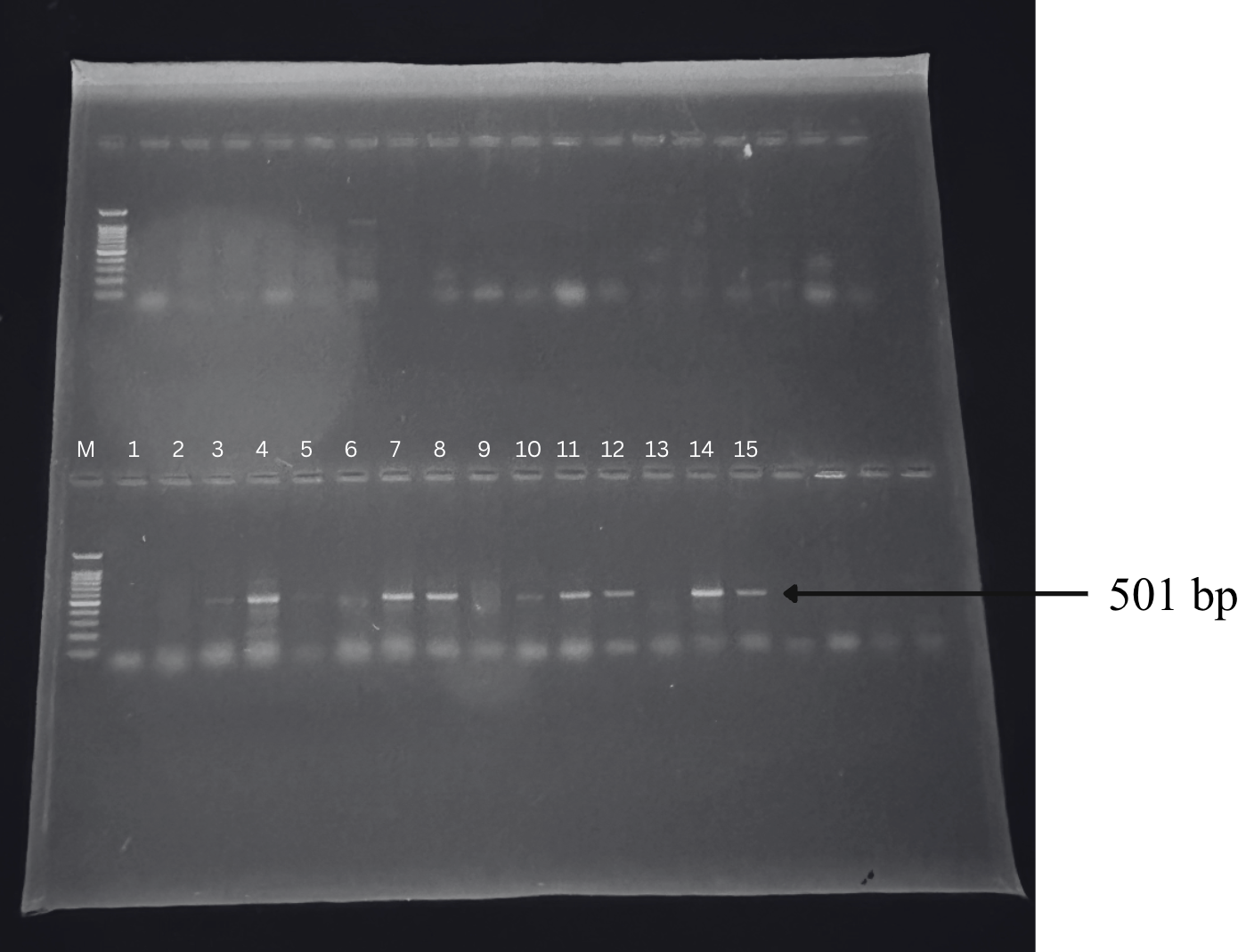

Supplement: S23 Fig — Here, Lane (M) is a 100 bp DNA marker, and Lane (3, 4, 7, 8, 10, 11, 12, 14, 15) are some positive samples at 501 bp. (TIF) [file pone.0336888.s026.tif]
