# Supplementary material for: Microbiological quality assessment of potential pathogenic bacteria and multidrug resistance patterns in commercial electrolyte drinks in Dhaka, Bangladesh
Source: PLoS One. 2026 Jun 2;21(6):e0336888. doi: 10.1371/journal.pone.0336888 (PMC13229343; doi:10.1371/journal.pone.0336888)
Supplement: Attachment — Submitted filename: Response to Reviewers.docx [file pone.0336888.s028.docx]

Comments to the Author:

1. Is the manuscript technically sound, and do the data support the conclusions? The manuscript must describe a technically sound piece of scientific research with data that supports the conclusions. Experiments must have been conducted rigorously, with appropriate controls, replication, and sample sizes. The conclusions must be drawn appropriately based on the data presented.

Reviewer #1: No

Reviewer #2: Yes

**Author’s response: We thank Reviewer #2 for their positive and constructive evaluation of our manuscript. We appreciate the reviewer’s recognition that the study represents a technically sound piece of scientific research, with data that appropriately support the conclusions drawn. All experiments were conducted rigorously, incorporating appropriate controls, replication, and sample sizes. Each experiment was performed in triplicate to ensure reproducibility and reliability of the results. Bacterial isolation and identification were carried out using widely accepted, commercially available culture media that are routinely employed in microbiological research and have been extensively reported in the literature. All experimental procedures followed standard microbiological methods and relevant guidelines. We are grateful for the reviewer’s acknowledgment of these aspects and for their overall assessment of the methodological robustness of our study.**

2. Has the statistical analysis been performed appropriately and rigorously?

Reviewer #1: No

Reviewer #2: Yes

**Author’s response: Yes, the statistical analyses performed in this study were appropriate for the descriptive and comparative nature of the data. The statistical analyses were conducted using widely accepted methods that have been extensively reported in the literature. Percentage-based analysis was used to summarize microbiological findings, including prevalence of bacterial contamination, antimicrobial resistance profiles, multidrug resistance, and MAR index values. These calculations were performed using Microsoft Excel, which is commonly used for descriptive statistical analysis in microbiological studies.**

**For the serum resistance assay, statistical significance was evaluated using a two-tailed test in Microsoft Excel to compare survival outcomes, and the corresponding p-values were calculated accordingly. All experiments were conducted in triplicate, and the resulting data were expressed as percentages or proportions to ensure reproducibility and clarity of interpretation. So, we are confident that we performed the statistical analysis appropriately.**

3. Have the authors made all data underlying the findings in their manuscript fully available?

The PLOS Data policy requires authors to make all data underlying the findings described in their manuscript fully available without restriction, with rare exception (please refer to the Data Availability Statement in the manuscript PDF file). The data should be provided as part of the manuscript or its supporting information or deposited to a public repository. For example, in addition to summary statistics, the data points behind means, medians and variance measures should be available. If there are restrictions on publicly sharing data—e.g. participant privacy or use of data from a third party—those must be specified.

Reviewer #1: No

Reviewer #2: Yes

**Author’s response: All data supporting the findings of this study are included within the manuscript. In addition, the raw antibiotic susceptibility testing (AST) data and serum resistance data for individual bacterial isolates have been uploaded as an Excel file named as “Supporting Datasheet,” along with all other associated files.**

4. Is the manuscript presented in an intelligible fashion and written in standard English?

PLOS ONE does not copyedit accepted manuscripts, so the language in submitted articles must be clear, correct, and unambiguous. Any typographical or grammatical errors should be corrected at revision, so please note any specific errors here.

Reviewer #1: No

Reviewer #2: Yes

**Author’s response: Reviewer #2 suggested that the manuscript was presented in an intelligible fashion and written in standard English. However, as Reviewer #1 disagreed with it, hence we rechecked the whole manuscript thoroughly by our colleague who is very fluent in English. Additionally, the language of the revised draft has been corrected using Grammarly Premium where it was relevant to make the manuscript easier to understand.**

5. Review Comments to the Author

Please use the space provided to explain your answers to the questions above. You may also include additional comments for the author, including concerns about dual publication, research ethics, or publication ethics. (Please upload your review as an attachment if it exceeds 20,000 characters)

Reviewer #1: The authors conducted this study to determine the presence and antimicrobial resistance profiles of various Gram-positive and Gram-negative bacteria isolated from local electrolytes in Bangladesh. This study seems interesting, but I found a lot of serious drawbacks! I believe the authors should check the CLSI guidelines properly before selecting antibiotics against different bacteria. Moreover, I found some serious issues in the AMR results. For example, *Klebsiella pneumoniae* is intrinsically resistant to ampicillin, amoxicillin, amoxicillin-clavulanic acid, and cefoxitin, but they found 30.77%, 19.23%, 3.85%, and 15.38% of the isolates, respectively. This result doesn’t make sense. The same happened to *Pseudomonas aeruginosa, Acinetobacter baumannii*, and others. Moreover, you mustn’t follow CLSI 2018 for *Vibrio* isolates; you should follow CLSI 2015. Also, you used the disk diffusion test for vancomycin, but you mustn’t do that. You must determine MIC values for *Staphylococcus aureus* against vancomycin to determine their resistance profiles. Overall, I feel the authors didn’t conduct the study properly. They should repeat the study with a proper study design and antimicrobial agent selection. They should also properly evaluate the lab work and results to maintain scientific integrity.

**Author’s response: We appreciate the reviewer’s comments. The reviewer mentioned that *Klebsiella pneumoniae* is intrinsically resistant to ampicillin, amoxicillin, amoxicillin-clavulanic acid, and cefoxitin. However, it is not entirely true because according to the Clinical and Laboratory Standards Institute (CLSI) guideline [1], *Klebsiella pneumoniae* is intrinsically resistant to only ampicillin and amoxicillin among the mentioned antibiotics by the reviewer. Nevertheless, there are reports where varying resistance were observed against these antibiotics. As a reference, we have added few papers where they found that the resistance of *Klebsiella pneumoniae* against these four antibiotics varies [2-5].**

**The reviewer mentioned that *Pseudomonas aeruginosa* and *Acinetobacter baumannii* are both intrinsically resistant to ampicillin, amoxicillin, amoxicillin-clavulanic acid, and cefoxitin. However, it is not entirely true because according to the Clinical and Laboratory Standards Institute (CLSI) guideline [1], *Pseudomonas aeruginosa* and *Acinetobacter baumannii* both are intrinsically resistant to ampicillin, ampicillin, and amoxicillin-clavulanic acid. Although resistance is common, there are documented cases in which a minority of isolates remain susceptible to cefoxitin [6,7].**

**For *Vibrio* spp., we rechecked the guideline and corrected the data according to the CLSI 2015 guideline.**

**For vancomycin, we agree that we should perform the MIC and not disc diffusion. However, we work in a resource limited lab, and with our current setup, although we wanted and we tried, it was not possible for us to perform MIC within a very short timeframe. Nevertheless, there are several reports where people still use disc diffusion for vancomycin [8,9]. Although, we agree that it will not give any conclusive result. However, it can give some ideas. Hence, we have carefully evaluated all reviewer comments. Although we do not concur with every point raised, therefore, we have revised the manuscript where appropriate to address the reviewer’s concerns and the changes are done in the manuscript with track changes on.**

Reviewer #2: The article describes the "Microbiological quality assessment of potential pathogenic bacteria and multidrug resistance patterns in commercial electrolyte drinks in Dhaka, Bangladesh". The article is well written with sufficient data and the results are convincing. However, the author should consider the following points before finalizing the manuscript.

1. The author isolated a number of bacteria from different samples. the author should add the images of grams staining as well as the characteristic colony of the isolated bacteria in the supplementary materials.
**Author’s response: We appreciate the reviewer’s valuable feedback. In response, we have added the images of Grams staining in the ‘S1 File’ (S1 Fig – S8 Fig) as well as the colony characteristic of the isolated bacteria (S1 Table).**

2. It would be good to show Antimicrobial Resistance (AMR) using heat maps, and this is a widely used and effective method in scientific research and public health surveillance. Heat maps provide a clear visual representation of complex AMR data, allowing researchers and public health officials to identify patterns, trends, and correlations easily.

**Author’s response: We are thankful for the constructive comment. We have provided the heatmap of the antimicrobial resistance (Fig 3) with associated description in the revised manuscript from line number (280 - 300).**

3. Discuss the production volume and popularity of electrolyte drink in Bangladesh in the introduction section of the manuscript.

**Author’s response: Electrolyte drinks have grown in popularity in Bangladesh's beverage market in recent years, especially in urban areas, where they are marketed as practical hydration options for fitness enthusiasts, outdoor workers, and consumers in general. Reliable data on production volume and popularity of electrolyte drinks are not publicly available because these items were sold for a long time without official authorization from the appropriate regulatory organizations. Only 2 out of the 9 tested electrolyte drink products have lately received regulatory approval, although the samples used in this study were gathered before these certifications. We found one online news portal-based article [10] and one national public newspaper (The Daily Star) article [11] where these issues were raised. However, between these two articles, The Daily Star is a well-established, accepted and recognized newspaper. So, it has credibility and authenticity. Which is why we have added this reference in the main manuscript. However, we have concerns regarding the credibility and authenticity of the online news article [8], which is why, even though we mentioned it here, we haven’t included it to the main manuscript. As a result, this study offers an evaluation of the microbiological quality of electrolyte drinks within a period of little regulatory supervision, highlighting the necessity of systematic surveillance and product-specific safety requirements as this beverage category develops.**

4. The authors identified antibiotic resistance only phenotypically, the authors can target known genes responsible for that resistance mechanism.

For Methicillin-resistant Staphylococcus aureus (MRSA): The primary target is the mecA or mecC gene, which encodes penicillin-binding protein 2a (PBP2a), conferring resistance to methicillin and most beta-lactams.

For Extended-Spectrum Beta-Lactamase (ESBL) producers (common in E. coli and Klebsiella): Common genes to screen for include blaCTX-M, blaTEM, and blaSHV subclasses.

For Carbapenem-resistant Gram-negative bacteria: Key carbapenemase genes include blaKPC, blaNDM, blaVIM, blaIMP, and blaOXA-48-like.

For Tetracycline resistance: Common genes are tetA, tetB, tetM, etc.

Data regarding the resistance mechanism will improve the quality of the manuscripts and would be more acceptable to the readers.

**Author’s response: We appreciate the reviewer's insightful recommendation. In response, the revised manuscript now includes a more thorough molecular examination of antibiotic resistance genes. We used PCR to identify important resistance genes related with the phenotypic resistance seen in our isolates, such as *mecA* (for MRSA), ESBL-associated genes (*bla_CTX-M_, bla_TEM_,* and *bla_SHV_*), and carbapenemase genes (*bla_KPC_, bla_NDM_,* and *bla_VIM_)*. These analyses' findings are now included in the “Results” section of the revised manuscript, where they are shown in a table (Table 3), with corresponding discussion included in the “Discussion” section.**

**We were unable to perform PCR for rest the genes, including *mecC, blaIMP*, *bla_OXA-48_*-like, and tetracycline resistance genes (*tetA, tetB, tetM*, etc.) due to limited resources. Despite this limitation, the tested genes are routinely employed in comparable food and environmental monitoring investigations and reflect the most frequently identified and clinically significant resistance genes for both Gram-positive and Gram-negative bacteria. We believe that this additional data regarding the resistance mechanism will improve the quality of our manuscript.**

**References**

1. Mathers AJ MD. Clinical and Laboratory Standards Institute [Internet]. Available from: <https://clsi.org/shop/standards/m100/>
2. Okafor JU, Nwodo UU. Molecular Characterization of Antibiotic Resistance Determinants in Klebsiella pneumoniae Isolates Recovered from Hospital Effluents in the Eastern Cape Province, South Africa. Antibiotics [Internet]. 2023 Jul 1;12(7):1139. Available from: <https://pubmed.ncbi.nlm.nih.gov/37508235/>
3. Worku M, Belay S, Molla T, Aynalem M, Assefa M. Prevalence and antimicrobial susceptibility pattern of Klebsiella pneumoniae isolated from various clinical specimens at the University of Gondar Comprehensive Specialized Hospital, Northwest Ethiopia. BMC Infectious Diseases [Internet]. 2024 Sep 4;24(1):917. Available from: <https://pmc.ncbi.nlm.nih.gov/articles/PMC11373452/>
4. Lin WP, Wang JT, Chang SC, Chang FY, Fung CP, Chuang YC, et al. The Antimicrobial Susceptibility of Klebsiella pneumoniae from Community Settings in Taiwan, a Trend Analysis. Scientific Reports [Internet]. 2016 Nov 8;6(1):36280. Available from: <https://pmc.ncbi.nlm.nih.gov/articles/PMC5099973/>
5. Wi YM, Choi JY, Lee DE, Jun SH, Kwon KT, Ko KS. Antimicrobial activity of cephamycins and β-lactam/β-lactamase inhibitors against ESBL-producing Escherichia coli and Klebsiella pneumoniae under standard and high bacterial inocula. Scientific Reports [Internet]. 2025 Mar 21;15(1):9785. Available from: <https://doi.org/10.1038/s41598-025-90762-1>
6. Mengestu AA, Semie NS, Tewabe DS. Bacteremia and antimicrobial resistance among febrile under five children attending pediatric clinic in Bahir Dar, Northwest Ethiopia: a retrospective study (2018–2024). International Journal of Scientific Reports [Internet]. 2025 Oct 23;11(11):387–93. Available from: <https://doi.org/10.18203/issn.2454-2156.intjscirep20253324>
7. Kombade, Sarika Prabhakar1,; Kaur, Navneet1; Patro, Sourabha Kumar2; Nag, Vijaya Lakshmi1. Clinico-bacteriological and antibiotic drug resistance profile of chronic suppurative otitis media at a tertiary care hospital in Western Rajasthan. Journal of Family Medicine and Primary Care 10(7):p 2572-2579, July 2021. | DOI: 10.4103/jfmpc.jfmpc_2480_20
8. Salah NM, Saafan AE, Salem EH, Rabey H a. E, Alsieni MA, Alatawi FA, et al. Inhibition of the Vancomycin Resistance in Staphylococcus aureus in Egypt Using Silver Nanoparticles. BioMed Research International [Internet]. 2022 Jan 1;2022(1):7380147. Available from: <https://doi.org/10.1155/2022/7380147>
9. Antimicrobial susceptibility pattern of clinical isolates of methicillin resistant Staphylococcus aureus [Internet]. PubMed. 2007. Available from: <https://pubmed.ncbi.nlm.nih.gov/17319409/>
10. Subscriber. How Bangladeshi companies selling electrolyte drinks without approval [Internet]. Available from: <https://www.dailycountrytodaybd.com/story/how-bangladeshi-companies-selling-electrolyte-drinks-without-approval>
11. Tayeb T. How are electrolyte drinks regulated in Bangladesh? The Daily Star [Internet]. Available from: <https://www.thedailystar.net/opinion/views/closer-look/news/how-are-electrolyte-drinks-regulated-bangladesh-3614591>
