# Supplementary material for: Microbiological quality assessment of potential pathogenic bacteria and multidrug resistance patterns in commercial electrolyte drinks in Dhaka, Bangladesh
Source: PLoS One. 2026 Jun 2;21(6):e0336888. doi: 10.1371/journal.pone.0336888 (PMC13229343; doi:10.1371/journal.pone.0336888)
Supplement: Attachment — Submitted filename: PONE-D-25-58950 Reviewers Comments.docx [file pone.0336888.s029.docx]

Microbiological quality assessment of potential pathogenic bacteria and multidrug resistance patterns in commercial electrolyte drinks in Dhaka, Bangladesh

Reviewer’s Comments:

1. Are all the local electrolyte drinks available made without BSTI approval? If not, it would help readers to know which brand of drinks are sampled for this study.
2. The introduction section is too long, could be helped from being precise and to the point.
3. Are the samples analyzed in triplicate to ensure statistical relevance?
4. APC protocol for juice sample followed could help from a relevant reference (Line 165).
5. Not all pathogenic bacteria are hemolytic. The study could help from performing further assays or molecular detection to identify different virulence factors in non-hemolytic isolates.
6. Table 1 is confusing. Column headers does not make sense with the data.
7. Narrow spectrum antibiotics like vancomycin and linezolid should not be included in the same percentage graph as the rest (Fig 2). To give an accurate representation through percentage, only antibiotics that have been applied on all 110 isolates should be included.
8. Please write scientific names in Italic font in the images.
9. The methods section should include the criteria by which an isolate is considered as multidrug resistant with reference.
10. The author should not repeat multidrug resistance everywhere, the abbreviation MDR is enough.
11. How was the MAR index calculated? The formula and interpretation should be included in the methods section.
12. The PCR primer list and the genes investigated should be included in the methods section instead as a supplementary file.
13. The method section does not mention the purpose of investigation of any ARGs and their target organisms. This should be included.
14. Methicillin is not listed as an antibiotic used in this study, and none of the result states so. How did the author investigate MRSA? Which isolates were considered as MRSA and investigated for mecA? Does harboring mecA makes a Staph isolate MRSA? Please clarify.
15. Gene names should be written using proper nomenclature, i.e., capital letter, italics etc.
16. Why weren’t the staph isolates subjected to the ESBL and MBL detection?
17. The discussion section in huge. It feels like going through the detailed discussion found in a thesis paper. Can it be reduced and remodeled so it can fit the precise and structured narrative of a manuscript?
