# Supplementary material for: Microbiological quality assessment of potential pathogenic bacteria and multidrug resistance patterns in commercial electrolyte drinks in Dhaka, Bangladesh
Source: PLoS One. 2026 Jun 2;21(6):e0336888. doi: 10.1371/journal.pone.0336888 (PMC13229343; doi:10.1371/journal.pone.0336888)
Supplement: Attachment — Submitted filename: Response to the Reviewers.docx [file pone.0336888.s030.docx]

Comments to the Authors:

1. Are all the local electrolyte drinks available made without BSTI approval? If not, it would help readers to know which brand of drinks are sampled for this study.

**Author’s response: Recently, only 2 out of the 9 tested electrolyte drink products have lately received regulatory approval from BSTI, although the samples used in this study were gathered before these certifications. The electrolyte samples analyzed in this study were commercially available products collected from local markets and most of them are well known companies. However, due to privacy and regulatory issues, we could not disclose the names.**

2. The introduction section is too long, could be helped from being precise and to the point.

**Author’s response: We appreciate the reviewer’s comments. The introduction has been substantially shortened and restructured to focus on (i) the rising consumption of electrolyte drinks, (ii) existing knowledge gaps in their microbiological safety, and (iii) the rationale and objectives of the present study. Redundant background information has been removed, reducing the section. The revised introduction now appears in lines (43-151).**

3. Are the samples analyzed in triplicate to ensure statistical relevance?

**Author’s response: Yes, all the samples and experiments were performed in triplicate to ensure reproducibility and reliability of the results, and reported values represent the mean of three independent experiments. We have now explicitly stated this in the Materials and Methods section (Line 225).**

4. APC protocol for juice sample followed could help from a relevant reference (Line 165).

**Author’s response: We thank the reviewer for this suggestion. The APC protocol followed in this study has now been cited with the relevant reference in line 233.**

5. Not all pathogenic bacteria are hemolytic. The study could help from performing further assays or molecular detection to identify different virulence factors in non-hemolytic isolates.

**Author’s response: We completely agree with the reviewer’s comment that hemolysis is one of the pathogenicity characteristics and there are many pathogenic isolates who do not lyse the red blood cells but have other virulence factors. That’s why, we just performed the hemolysis test to identify about what percentage of our isolates can lyse the red blood cells and about performing further assays or molecular detection to identify different virulence factors in non-hemolytic isolates, because of resource limitation, though we wanted, it was not possible for us to check other virulence factors by molecular detection. Still, we are trying if we can perform other virulence tests or not, but in our current resource limited setup, it will be very time consuming. And, if we wait for long, it might be late to inform people about this public health related issues regarding electrolyte drinks. For this reason, we wanted individuals to be aware of the current information we have gathered.**

6. Table 1 is confusing. Column headers does not make sense with the data.

**Author’s response: We thank the reviewer for pointing out this issue. Table 1 has been reformatted with clearer, more understandable column headers. However, as we have included a new table in the manuscript, now Table 1 in the revised manuscript is renamed as “Table 2.”**

7. Narrow spectrum antibiotics like vancomycin and linezolid should not be included in the same percentage graph as the rest (Fig 2). To give an accurate representation through percentage, only antibiotics that have been applied on all 110 isolates should be included.

**Author’s response: We thank the reviewer for this important methodological point. We agree that combining antibiotics tested only against Gram-positive isolates (such as vancomycin and linezolid) with broad-spectrum antibiotics applied to all 110 isolates produces a misleading percentage representation. Figure 2 has been divided into two parts (a and b). Part a representing the resistance observed against broad spectrum antibiotics and part b representing the resistance observed against the Gram-positive antibiotics. Moreover, considering the intrinsic resistance of different organism against different antibiotics, we cannot apply all the antibiotics on all 110 isolates. Which is why, the isolates with intrinsic resistance were not included in the percentage analysis.**

8. Please write scientific names in Italic font in the images.

**Author’s response: All scientific names in Figures 3 and 4 have been corrected to italicized formatting. The figure captions have been similarly revised throughout the manuscript.**

9. The methods section should include the criteria by which an isolate is considered as multidrug resistant with reference.

**Author’s response: We have added the formal definition of multidrug resistance to the Materials and Methods section in lines (234-236) with relevant reference.**

10. The author should not repeat multidrug resistance everywhere, the abbreviation MDR is enough.

**Author’s response: We thank the reviewer for this suggestion. The full term "multidrug resistance" has been retained only at its first mention (where the abbreviation is defined), and MDR has been used consistently throughout the rest of the manuscript.**

11. How was the MAR index calculated? The formula and interpretation should be included in the methods section.

**Author’s response: We have now added the MAR index calculation formula and interpretation to the Materials and Methods section (Line 278-283).**

12. The PCR primer list and the genes investigated should be included in the methods section instead as a supplementary file.

**Author’s response: As recommended, the full list of PCR primers, target genes, and amplicon sizes has been moved from the supplementary file into the main Materials and Methods section as Table 1.**

13. The method section does not mention the purpose of investigation of any ARGs and their target organisms. This should be included.

**Author’s response: We thank the reviewer for highlighting this omission. We have added a paragraph in the Materials and Methods section (Line 288-292) explaining the rationale for investigating each antibiotic resistance gene (ARG) and the corresponding target organisms.**

14. Methicillin is not listed as an antibiotic used in this study, and none of the result states so. How did the author investigate MRSA? Which isolates were considered as MRSA and investigated for mecA? Does harboring mecA makes a Staph isolate MRSA? Please clarify.

**Author’s response: We couldn’t check the phenotypic resistance of methicillin unfortunately. However, in line with current CLSI (M100, 2024) guidelines, cefoxitin (30 µg) disk diffusion was used as the phenotypic screening method for methicillin resistance as it is considered as surrogate marker for *mecA* mediated resistance. Isolates with cefoxitin zone diameters ≤21 mm were considered methicillin-resistant and subjected to *mecA* PCR for molecular confirmation. Detection of the *mecA* gene alone, without phenotypic confirmation of methicillin resistance, is insufficient for definitive MRSA classification, according to CLSI 2024 [1]; such isolates are more accurately designated as putative MRSA.**

15. Gene names should be written using proper nomenclature, i.e., capital letter, italics etc.

**Author’s response: All gene names have been corrected to follow standard nomenclature conventions throughout the manuscript.**

16. Why weren’t the staph isolates subjected to the ESBL and MBL detection?

**Author’s response: We thank the reviewer for this question. To the best of our knowledge and based on current literature, *Staphylococcus* species do not characteristically harbor ESBL or MBL encoding genes. ESBL and MBL detection assays are standardized and validated primarily for Gram-negative organisms [2,3], particularly *Enterobacterales* and non-fermenters, since these organisms are the predominant producers of these β-lactamases. Staphylococcus species do not characteristically harbor ESBL- or MBL-encoding genes (*bla_CTX-M_, bla_TEM_, bla_SHV_, bla_NDM_, bla_VIM_, bla_KPC_*); their β-lactam resistance is mediated through fundamentally different mechanisms (notably *mecA/mecC*-encoded altered PBP2a and *bla_Z_*-encoded penicillinase) [4,5]. Including *Staphylococcus* isolates in ESBL/MBL screening would therefore not yield biologically meaningful results.**

17. The discussion section in huge. It feels like going through the detailed discussion found in a thesis paper. Can it be reduced and remodeled so it can fit the precise and structured narrative of a manuscript?

**Author’s response: We fully agree. The Discussion has been substantially condensed and restructured to fit the format of a research manuscript. Redundant explanations, repeated context, and overly detailed elaborations have been removed, while all key findings, comparisons with prior literature, and references have been preserved. The revised Discussion appears in Lines 479–977, with all changes visible in the tracked-changes version of the manuscript.**

**References**

1. Clinical and Laboratory Standards Institute. Performance Standards for Antimicrobial Susceptibility Testing. 34th ed. CLSI supplement M100. Wayne, PA: Clinical and Laboratory Standards Institute; 2024.
2. Castanheira M, Simner PJ, Bradford PA. Extended-spectrum β-lactamases: an update on their characterization, epidemiology and detection. JAC Antimicrob Resist. 2021;3(3):dlab092.
3. Wu W, Feng Y, Tang G, Qiao F, McNally A, Zong Z. NDM Metallo-β-Lactamases and Their Bacterial Producers in Health Care Settings. Clin Microbiol Rev. 2019;32(2):e00115-18.
4. Llarrull LI, Fisher JF, Mobashery S. Molecular basis and phenotype of methicillin resistance in *Staphylococcus aureus* and insights into new β-lactams that meet the challenge. Antimicrob Agents Chemother. 2009;53(10):4051–4063.
5. Lade H, Kim JS. Molecular determinants of β-lactam resistance in methicillin-resistant *Staphylococcus aureus* (MRSA): an updated review. Antibiotics (Basel). 2023;12(9):1362.
